# RNA decay is an antiviral defense in plants that is counteracted by viral RNA silencing suppressors

**DOI:** 10.1371/journal.ppat.1007228

**Published:** 2018-08-03

**Authors:** Fangfang Li, Aiming Wang

**Affiliations:** London Research and Development Centre, Agriculture and Agri-Food Canada, London, Ontario, Canada; University of Cambridge, UNITED KINGDOM

## Abstract

Exonuclease-mediated RNA decay in plants is known to be involved primarily in endogenous RNA degradation, and several RNA decay components have been suggested to attenuate RNA silencing possibly through competing for RNA substrates. In this paper, we report that overexpression of key cytoplasmic 5’–3’ RNA decay pathway gene-encoded proteins (5’RDGs) such as decapping protein 2 (DCP2) and exoribonuclease 4 (XRN4) in *Nicotiana benthamiana* fails to suppress sense transgene-induced post-transcriptional gene silencing (S-PTGS). On the contrary, knock-down of these 5’RDGs attenuates S-PTGS and supresses the generation of small interfering RNAs (siRNAs). We show that 5’RDGs degrade transgene transcripts via the RNA decay pathway when the S-PTGS pathway is disabled. Thus, RNA silencing and RNA decay degrade exogenous gene transcripts in a hierarchical and coordinated manner. Moreover, we present evidence that infection by turnip mosaic virus (TuMV) activates RNA decay and 5’RDGs also negatively regulate TuMV RNA accumulation. We reveal that RNA silencing and RNA decay can mediate degradation of TuMV RNA in the same way that they target transgene transcripts. Furthermore, we demonstrate that VPg and HC-Pro, the two known viral suppressors of RNA silencing (VSRs) of potyviruses, bind to DCP2 and XRN4, respectively, and the interactions compromise their antiviral function. Taken together, our data highlight the overlapping function of the RNA silencing and RNA decay pathways in plants, as evidenced by their hierarchical and concerted actions against exogenous and viral RNA, and VSRs not only counteract RNA silencing but also subvert RNA decay to promote viral infection.

## Author summary

RNA silencing and RNA decay are two essential pathways that determine the fate of cellular RNA molecules. RNA decay has been suggested to compete with RNA silencing for the RNA substrates and thus to suppress RNA silencing in plants. In this report, we show that the deficiency of key cytoplasmic 5’–3’ RNA decay pathway gene-encoded proteins (5’RDGs) rather than overexpression of them attenuates RNA silencing and facilitates viral RNA accumulation in turnip mosaic virus (TuMV)-infected *Nicotiana benthamiana* plants. 5’RDGs can inhibit the accumulation of exogenous gene transcripts or viral RNA via the RNA decay pathway when RNA silencing is compromised. Therefore, RNA silencing and RNA decay are important defense mechanisms that coordinate to function against exogenous gene transcripts or viral RNA. It is well known that viruses have evolved to encode viral suppressors of RNA silencing (VSRs) that counteract RNA silencing for an invading virus to establish infection. We discover that two known VSRs of TuMV interact with 5’RDGs and the interactions subvert RNA decay. Taken together, this study provides new insights into the concerted function of RNA silencing and RNA decay in plant immunity against exogenous gene transcripts and viral RNA, and reveals a novel function of VSRs.

## Introduction

Among major infectious pathogens, viruses are the obligate intracellular agents that can infect all types of life forms from bacteria to plants and animals, and exclusively multiply in the host cells. The vast majority of known plant viruses are positive-sense single-stranded (+ss) RNA viruses that typically have a relatively small genome encoding no more than a dozen proteins. Since the genomic RNAs of +ss viruses are similar to the endogenous mRNAs, the regulation machinery of cellular RNA metabolism in the infected plants is unavoidably involved in viral infection.

An essential RNA regulation mechanism is RNA silencing, which is an evolutionarily conserved and sequence-specific immunity response and triggered by double-stranded RNAs (dsRNAs) [1–3]. In plants, RNA silencing is recognized as a central antiviral pathway [2,3]. During viral infection, dsRNAs may originate from viral genome replication, via discrete intramolecular pairing within viral genomic RNA or *de novo* synthesis by the endogenous RNA-dependent RNA polymerases (RdRps) [4]. These dsRNAs are processed into 21- to 24- nucleotide (nt) small interfering RNA (siRNA) duplexes by the dsRNA-specific Dicer-like (DCL) proteins that contain RNase III activity. The resulting siRNAs duplexes are incorporated into the RNA-induced silencing complex (RISC) and guide the sequence-specific degradation of the target viral RNA by the Argonaute (AGO) protein that possesses RNaseH-like endonuclease activity [4,5]. As a consequence of the co-evolutionary arms race between plants and viruses, viruses have evolved to suppress or evade RNA silencing by encoding viral suppressors of RNA silencing (VSRs). In the past twenty years, VSRs have been identified from almost all plant virus genera [5].

In addition to RNA silencing, RNA decay is another crucial pathway that regulates RNA turnover [6,7]. RNA decay or exonucleolytic RNA turnover is a 5’–3’ and 3’–5’ exoribonuclease-dependent, ubiquitous mechanism in eukaryotic cells by which mRNA molecules are enzymatically degraded [6]. RNA decay is essential for both mRNA quantity and quality control [6]. The degradation process is initiated by deadenylation to progressively remove the 3’ poly(A) tail followed by exosome complex-mediated 3’–5’ cleavage or decapping and subsequent exoribonuclease (XRN)-mediated 5’–3’ decay [6,7]. Deadenylation is catalysed by the conserved poly (A)-specific ribonuclease (PARN) as well as by the conserved carbon catabolite repressor 4 (CCR4) complex [8–10], and the removal of the 5’ cap structure is through concerted action of a set of conserved decapping proteins (DCP) [6,7]. In *Arabidopsis thaliana*, DCP1, DCP2, DCP5, VARICOSE (VCS) and possibly DEA(D/H)-box RNA helicase 1 (DHH1) constitute the decapping complex [11]. Deadenylation or decapping is a prerequisite for most RNA to be degraded by the 3’–5’ exoribonuclease exosome complex or by 5’–3’ XRN exoribonucleases, respectively [6,7]. Arabidopsis encodes three XRN proteins, AtXRN2, AtXRN3 and AtXRN4 [11,12]. AtXRN4 is a predominantly cytoplasmic exoribonuclease that co-localizes with decapping proteins to form plant-processing bodies (P-bodies) for 5’–3’ RNA decay [11], whereas functionally redundant XRN2 and XRN3 degrade transcripts within the nucleus [12].

Accumulated evidence suggests that RNA decay is also a major cellular antiviral mechanism [13]. Since viral RNAs are different for typical cellular mRNAs, the host RNA decay machinery can recognize and target these foreign RNAs for degradation. Indeed, knockout of key RNA decay pathway genes enhances viral infection and promotes the accumulation of viral RNA in infected cells [13]. A genome-wide screen of *Saccharomyces cerevisiae* single-gene deletion library and subsequent work revealed that XRN1p is involved in the degradation of tomato bushy stunt virus (TBSV) RNA in yeast [14,15]. Consistently, silencing of *NbXRN4* in *Nicotiana benthamiana*, homologous to *XRN1* in yeast, promotes viral RNA accumulation of tobacco mosaic virus (TMV) and TBSV [16,17]. The viral RNA level in Arabidopsis *dcp2* mutant plants infected by a recombinant tobacco rattle virus (TRV) carrying the *green fluorescence protein* gene (*GFP*) is significantly higher than that in wild type plants [18]. In contrast, ectopic overexpression of *AtXRN4* from Arabidopsis and *OsXRN4* from *Oryza sativa* in *N*. *benthamiana* suppresses cucumber necrosis virus (CNV) and TMV infection, respectively [15,19]. Then, how do viruses cope with RNA decay? In virus-infected metazoan cells, viruses manage to repress the key aspects of the host RNA decay pathway, which prevents the degradation of viral genomic RNA and promotes viral infection [13]. For instance, hepatitis C virus (HCV) targets the P-bodies and recruits P-body proteins for viral genome replication [20–22]. In the cases of picornaviruses such as poliovirus and human rhinovirus, viral proteases and/or the host cell proteasome appear to be involved in the degradation of the essential RNA decay proteins [23,24].

Despite recent progress in understanding the antiviral role of RNA silencing and RNA decay, many fundamental questions are yet to be unanswered. For instance, it is not clear if these two pathways are interlinked to operate against viral infection and whether they function simultaneously, sequentially or preferentially. The molecular mechanism underlying virus-mediated suppression of RNA decay remains poorly understood. This is particularly true in virus-plant interactions. In this study, we used turnip mosaic virus (TuMV) and *N*. *benthamiana* as a model system to investigate the possible involvement of several essential cytoplasmic 5’–3’ RNA decay pathway genes (*5’RDGs*) in RNA silencing and RNA decay in virally infected plant cells. We found that knockdown of *5’RDGs* but not overexpression of them suppresses PTGS induced by sense transcripts, and RNA silencing, compared to RNA decay, plays a predominant role in the degradation of foreign transcripts (*GFP* or viral RNA). VSRs could hijack the key components from these pathways to suppress plant defense. These data highlight the overlapping function of the RNA silencing and RNA decay pathways in plants, as evidenced by their hierarchical and cooperative actions against foreign transcripts, and VSRs not only supress RNA silencing but also RNA decay to promote viral infection in plants.

## Results

### Sequence analysis, expression pattern and subcellular localization of NbDCP1, NbDCP2, NbXRN4 and NbPARN

**Fig 1:**
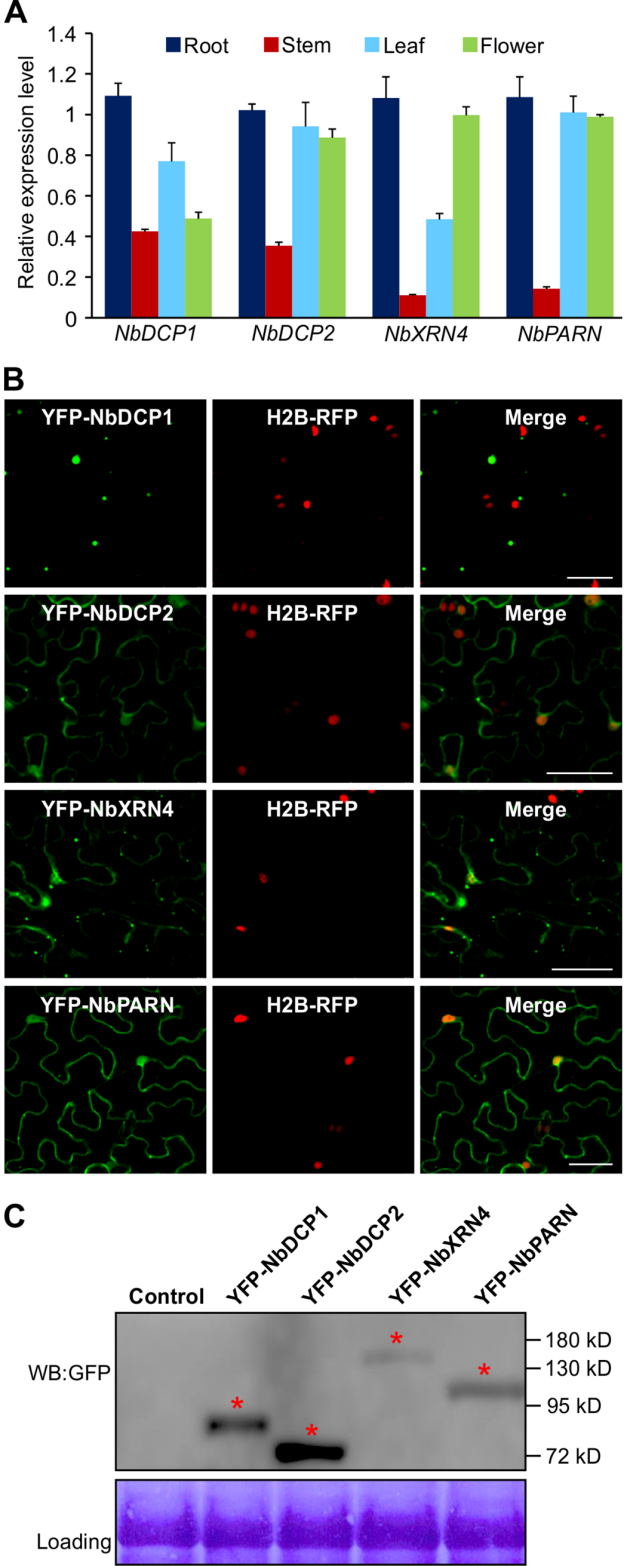
Molecular characterization of 5’RDGs. **(A)** qRT-PCR analysis of *NbDCP1*, *NbDCP2*, *NbXRN4* and *NbPARN* expression levels in different tissues of *N*. *benthamiana*. Expression was normalized against *NbActin* transcripts, which serve as an internal standard. Each mean value was derived from three independent experiments (n = 3 samples). Values represent the mean ± standard deviation (SD). **(B)** Micrographs showing cells from leaves of H2B-RFP transgenic *N*. *benthamiana* expressing YFP-NbDCP1, YFP-NbDCP2, YFP-NbXRN4 or YFP-NbPARN. Bars = 50 μm. (**C**) Western Blot (WB) analysis of total protein extracts from infiltrated leaves as indicated in (B) at 32 hours post infiltration (hpi), antibody against GFP (WB:GFP) was applied. Red asterisks indicate the expected band sizes. Coomassie brilliant blue-stained Rubisco large subunit was used as a loading control.

We initiated this study by cloning and sequencing of four key *5’RDGs* from *N*. *benthamiana*, including *NbDCP1*, *NbDCP2*, *NbXRN4* and *NbPARN*. Sequence analysis revealed that the open reading frames (ORFs) of *NbDCP1*, *NbDCP2*, *NbXRN4* and *NbPARN* contain 1113 nt (GenBank accession number: KY402210), 966 nt (GenBank accession number: KY402211), 2949 nt (GenBank accession number: KY402212), and 2056 nt (GenBank accession number: KY402213), respectively. The deduced amino acid sequences of NbDCP1, NbDCP2, NbXRN4 and NbPARN share 70.2%, 68.4%, 67.1% and 62% identity with their counterparts AtDCP1, AtDCP2, AtXRN4 and AtPARN from Arabidopsis, respectively. To document basic biology features of these 5’RDGs from *N*. *benthamiana*, we determined the expression pattern of these genes and subcellular localization of their encoded proteins. Quantitative real-time reverse-transcription PCR (qRT-PCR) was performed using total RNA isolated from different *N*. *benthamiana* tissues as template. All the four genes were constitutively expressed but their expression levels varied at different tissues (**Fig 1A**). Overall, all the four genes showed the highest expression level in the root tissue, and the lowest in the stem tissue.

To examine the subcellular localization of these four *N*. *benthamiana* 5’RDGs, their coding regions were firstly introduced into pDNOR221 vector by BP reaction (Invitrogen), followed by recombination into in frame downstream of the coding sequence of yellow fluorescence protein (YFP) by LR reaction (Invitrogen). The chimeric genes were transiently expressed in leaves of transgenic H2B-RFP *N*. *benthamiana* plants, and fluorescence was examined in agroinfiltrated transgenic leaves at 32 hours post infiltration (hpi) by confocal microscopy (**Fig 1B)**. YFP-NbDCP1 was present predominantly in the cytoplasm, forming the round granules, whereas the other three including YFP-NbDCP2, YFP-NbXRN4 and YFP-NbPARN were evident in both the cytoplasm and nucleus, and some of YFP-NbDCP2, YFP-NbXRN4 and YFP-NbPARN aggregated to form granules in the cytoplasm as well (**Fig 1B**). Western blotting using GFP (WB:GFP) antibody (**Fig 1C**) detected the expected and specific band corresponding to the YFP-tagged NbDCP1, NbDCP2, NbXRN4 and NbPARN protein, confirming the presence of full-length recombinant proteins.

### The interaction and co-localization of NbDCP1, NbDCP2 and NbXRN4

**Fig 2:**
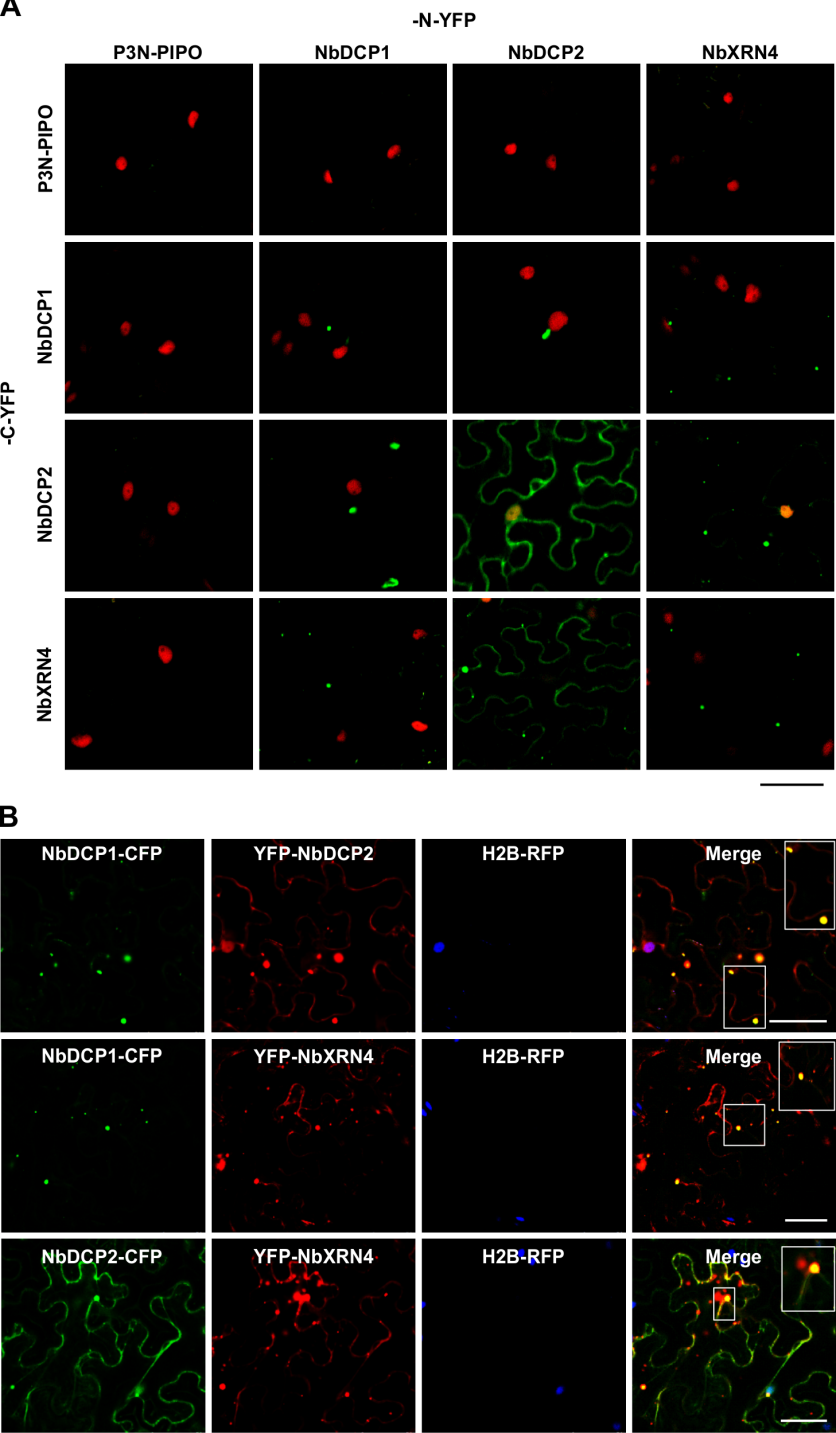
Interactions and subcellular co-localization of NbDCP1, NbDCP2 and NbXRN4. **(A)** BiFC assays between NbDCP1, NbDCP2 and NbXRN4 in H2B-RFP transgenic *N*. *benthamiana* leaves at 32 hpi. Yellow fluorescence (green) resulted from the interaction of two tested proteins tagged by the C-terminal half of YFP (C-YFP) or the N-terminal half of YFP (N-YFP). Nuclei of tobacco leaf epidermal cells are indicated by expression of the H2B-RFP transgene (red). P3N-PIPO tagged by C-YFP or N-YFP serves as a negative control. Bars = 50 μm. **(B)** Co-localization of NbDCP1, NbDCP2 and NbXRN4 in the H2B-RFP transgenic *N*. *benthamiana* leaf cells at 32 hpi. The yellow signals result from the overlapping of NbDCP1-CFP (green) with YFP-NbDCP2 (red) or YFP-NbXRN4 (red), or NbDCP2-CFP (green) with YFP-NbXRN4 (red). Insets are the enlarged images of the areas in white boxes in the corresponding panels. H2B-RFP is shown in blue. Bars = 50 μm.

To determine whether NbDCP1, NbDCP2 and NbXRN4 interact with each other, we performed bimolecular fluorescence complementation (BiFC) assays [25]. The N- and C-YFP tagged proteins were co-expressed in leaves of transgenic H2B-RFP *N*. *benthamiana* plants and analyzed by live cell fluorescence microscopy at 32 hpi (**Fig 2A**). Self as well as all mutual combinations of NbDCP1, NbDCP2, and XRN4 showed positive interactions. P3N-PIPO, which is a dedicated movement protein of TuMV [26], was fused to N- or C-YFP to serve a negative control (**Fig 2A**). The viral proteins P3N-PIPO and CI that interact to form a complex for viral cell-to-cell movement [27] were used as a positive control (**S1 Fig**). Interestingly, the NbDCP2 self-interacting complex was present in the cytoplasm as well in the nucleus. Except the NbDCP2 self-interacting complex and the NbDCP2-N-YFP and NbXRN4-C-YFP combination, both of which were evenly distributed in the cytoplasm, the remaining combinations all formed cytoplasmic foci, regardless of reciprocal fusion with an N- or C-YFP (**Fig 2A**).

We further conducted a co-localization assay to confirm whether theses interaction complexes are formed in the same protein complexes. C-terminal cyan fluorescent protein (CFP) fused NbDCP1 (NbDCP1-CFP) and N-terminal YFP fused NbDCP2 (YFP-NbDCP2) or NbXRN4 (YFP-NbXRN4), or C-terminal CFP fused NbDCP2 (NbDCP2-CFP) and YFP-NbXRN4 were co-expressed in the leaves of H2B transgenic *N*. *benthamiana* plants, and confocal microscopy was performed at 32 hpi. We found that NbDCP1-CFP and YFP-NbDCP2 or YFP-NbXRN4 co-localized in the cytoplasm to form the bright granules (**Fig 2B**). Such bright foci were also observed in the cytoplasm of cells co-expressing NbDCP2-CFP and YFP-NbXRN4 (**Fig 2B**). Taken together, these data suggest that NbDCP1, NbDCP2 and NbXRN4 can form protein complexes through protein-protein interactions.

### Overexpression of NbDCP1, NbDCP2, NbXRN4 or NbPARN fails to suppress GFP-induced RNA silencing

**Fig 3:**
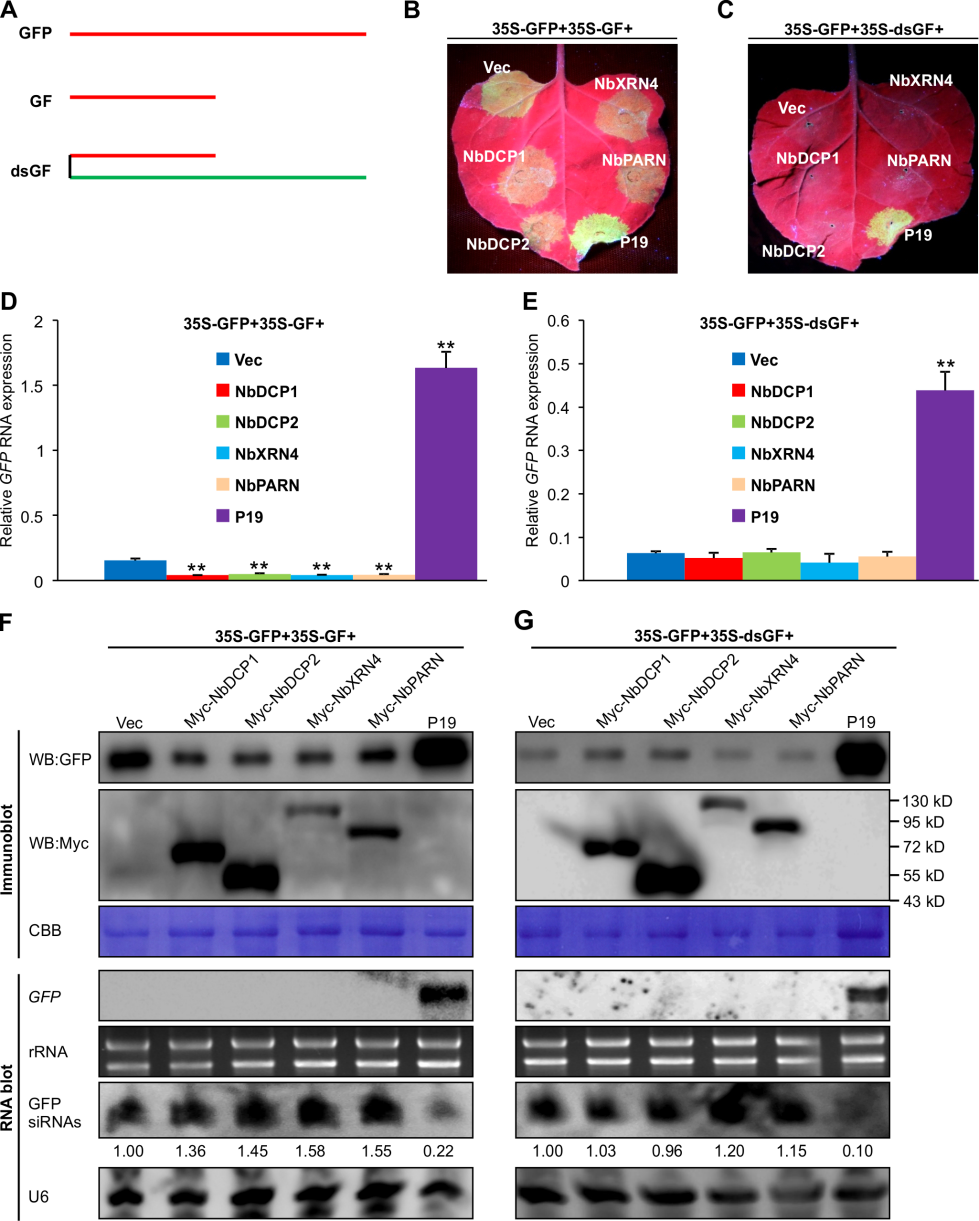
Expression of NbDCP1, NbDCP2, NbXRN4, or NbPARN fails to suppress GFP-induced RNA silencing in *N*. *benthamiana* plants. **(A)** Schematic representation of the RNA fragment derived from expression vectors GFP, GF and dsGF. (**B, C**) Pictures of representative agroinfiltrated leaves taken at 5 dpi under UV light. Leaf patches were agroinfiltrated with three vectors including 35S-GFP, 35S-GF (or 35S-dsGF) and one of the following vectors: an empty vector (Vec), Myc-tagged-NbDCP1, NbDCP2, NbXRN4, NbPARN, and TBSV p19 (B). Similar results were obtained from three independent experiments. (**D, E**) Analyses of relative accumulations of *GFP* mRNAs by specific qRT-PCR in the infiltrated leaves shown in (B, C) at 5 dpi. *NbActin* serves as an internal standard. Each mean value was calculated based on three independent experiments (n = 3 samples). Values represent the mean ± SD. Double asterisks indicate a highly significant difference compared to 35S-GFP+35S-GF+Vec (D) or 35S-GFP+35S-dsGF+Vec (E) (*P* < 0.01, Student’s *t* test). (**F, G**) Accumulations of GFP protein, Myc-tagged-NbDCP1, NbDCP2, NbXRN4, NbPARN protein, *GFP* mRNAs and siRNAs in the infiltrated leaves shown in (B, C) at 5 dpi. Protein levels were analyzed by immunoblot analysis using antibodies against GFP (WB:GFP) or Myc (WB:Myc). Coomassie brilliant blue (CBB) staining of the large subunit of Rubisco, ethidium bromide staining of rRNA, and U6 serve as a loading control for immunoblot, mRNA blot and siRNA blot, respectively. The values of GFP siRNAs/U6 were quantified by ImageJ software and then normalized against the mean value corresponding to the Vec treatment, which was set to 1.00.

Several recent studies have reported that essential components of the cytoplasmic 5’–3’ RNA decay pathway including XRN4 and DCP2 can suppress sense RNA-induced PTGS (S-PTGS), but not inverted repeat RNA-induced PTGS (IR-PTGS) in Arabidopsis, suggesting that the RNA silencing and 5’–3’ RNA decay pathways are interlinked, possibly by sharing the same RNA substrate [28,29]. Consistently, compromising nonsense-mediated decay, deadenylation or exosome activity enhances S-PTGS, which requires host RNA-dependent RNA polymerase 6 (RDR6) and SUPPRESSOR OF GENE SILENCING 3 (SGS3) for the transformation of single-stranded RNA into dsRNA to trigger PTGS [30,31]. To determine whether the *N*. *benthamiana* 5’RDGs also affect S-PTGS and IR-PTGS as those in Arabidopsis, *Agrobacterium* cultures expressing 35S-GFP, 35S-GF (GF: a fragment of GFP that can induce S-PTGS, **Fig 3A**) and N-Myc-tagged NbDCP1, NbDCP2, NbXRN4 or NbPARN were co-infiltrated into *N*. *benthamiana* leaves. Leaves co-infiltrated with 35S-GFP, 35S-GF and empty vector (Vec) or the vector for expression of TBSV P19 (a well-known gene silencing suppressor) serve controls. Agroinfiltration of *N*. *benthamiana* leaves with 35S-GFP, 35S-GF and Vec induced *GFP* RNA silencing, and led to reduced GFP fluorescence under UV light at 5 days post infiltration (dpi) (**Fig 3B**). While the intensity of green fluorescence increased substantially in leaf patches co-expressing GFP and P19, no obvious differences of fluorescence were observed among leaf patches co-expressing Myc-tagged NbDCP1, NbDCP2, NbXRN4 or NbPARN or co-infiltrated with the vector control (**Fig 3B**). Therefore, transient expression of the four 5’RDGs was unable to suppress the sense RNA trigger (35S-GF)-induced *GFP* silencing. On the contrary, expression of these 5’RDGs led to the generation of more GFP-derived siRNAs and the reduced levels of *GFP* mRNA and protein, enhancing sense GFP-induced RNA silencing (**Fig 3D and 3F)**. We also examined the possible effects of the four 5’RDGs on dsRNA-induced gene silencing. Similar to previous observations [28–30], there was no obvious RNA silencing suppression or enhancement, when 35S-dsGF (dsRNA of GF) as the silencing trigger co-expressed with 35S-GFP and N-Myc-tagged NbDCP1, NbDCP2, NbXRN4 or NbPARN in *N*. *benthamiana* leaves (**Fig 3C, 3E and 3G**). As a positive control, P19 suppressed S-PTGS and IR-PTGS, and intensified GFP fluorescence, which was confirmed by immunoblot and RNA blot analyses (**Fig 3**). Taken together these data demonstrate that the four *N*. *benthamiana* 5’RDGs are likely involved in GFP-induced S-PTGS, but not in IR-PTGS.

### Knock-down of *NbDCP1*, *NbDCP2*, *NbXRN4* and *NbPARN* inhibits S-PTGS, not IR-PTGS

**Fig 4:**
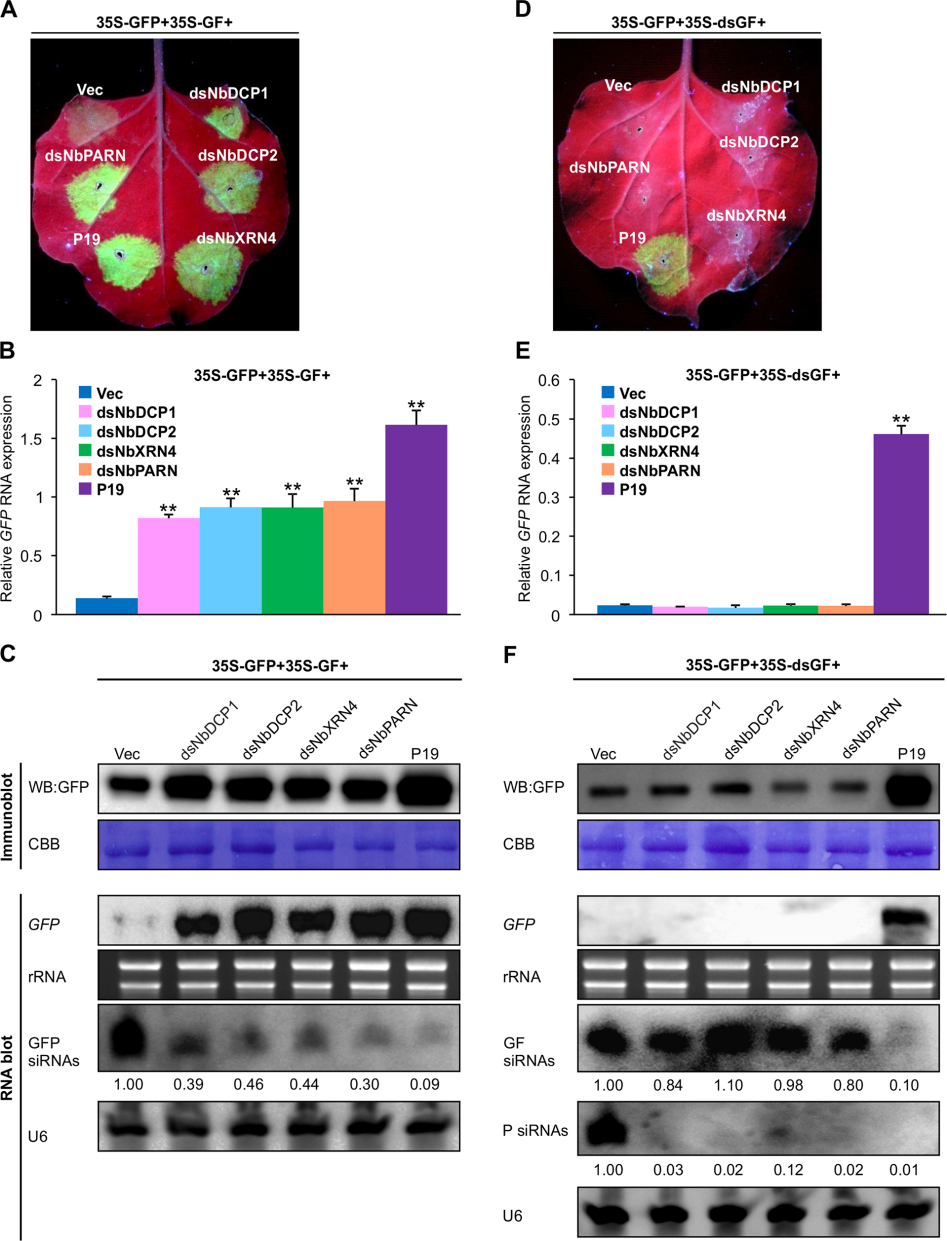
Silencing of *NbDCP1*, *NbDCP2*, *NbXRN4*, or *NbPARN* suppresses S-PTGS, but not IR-PTGS in *N*. *benthamiana* plants. (**A, D**) Visualization of green fluorescence of representative agroinfiltrated leaves. Leaf patches were agroinfiltrated with three expression vectors including 35S-GFP, 35S-GF (or 35S-dsGF) and one of the following vectors: an empty vector (Vec), dsNbDCP1, dsNbDCP2, dsNbXRN4, dsNbPARN, P19, Myc-tagged-NbDCP1, NbDCP2, NbXRN4, and NbPARN. Pictures were taken at 5 dpi under UV light. Similar results were obtained from three independent experiments. (**B, E**) Relative accumulation of *GFP* mRNAs in agroinfiltrated patches of the infiltrated leaves indicated in (A) and (D), respectively. Total RNA was isolated at 5 dpi and *GFP* mRNA was analyzed by qRT-PCR. *NbActin* serves as an internal standard. Each mean value was derived from three independent experiments (n = 3 samples). Values represent the mean ± SD. Double asterisks indicate a highly significant difference compared to 35S-GFP+35S-GF+Vec (B) or 35S-GFP+35S-dsGF+Vec (E) (*P* < 0.01, Student’s *t* test). (**C, F**) Accumulation of GFP protein, *GFP* mRNA and siRNAs in the infiltrated leaves shown in (A) and (D), respectively. Samples were collected at 5 dpi. CBB staining of the large subunit of Rubisco, ethidium bromide staining of rRNA, and U6 serve as a loading control for immunoblot, mRNA blot and siRNA blot, respectively. The values of GFP siRNAs/U6 were quantified by ImageJ software and then were normalized against the mean value corresponding to the Vec treatment, which was set to 1.00.

To further determine whether NbDCP1, NbDCP2, NbXRN4 or NbPARN is involved in RNA silencing, three expression vectors including 35S-GFP, 35S-GF and a hairpin RNAi construct containing NbDCP1 (dsNbDCP1), NbDCP2 (dsNbDCP2), NbXRN4 (dsNbXRN4) or NbPARN (dsNbPARN) sequences under the control of the cauliflower mosaic virus (CaMV) 35S promoter, were agroinfiltrated into leaves of *N*. *benthamiana* plants. Compared to the weak GFP fluorescence in *N*. *benthamiana* leaf patches infiltrated with 35S-GFP, 35S-GF and Vec, expression of dsNbDCP1, dsNbDCP2, dsNbXRN4 or dsNbPARN led to an increase in green fluorescence in co-infiltrated regions (**Fig 4A**), indicating that silencing of *NbDCP1*, *NbDCP2*, *NbXRN4* or *NbPARN* suppressed sense GFP-induced RNA silencing. Accordingly, qRT-PCR, RNA blot and protein gel blot analyses revealed that suppression of GFP silencing by dsNbDCP1, dsNbDCP2, dsNbXRN4 or dsNbPARN was accompanied by the increased levels of *GFP* mRNA and protein, and the reduced levels of GFP-specific siRNAs in the infiltrated leaves (**Fig 4B and 4C**). As a control, a hairpin RNAi construct of GUS failed to suppress S-PTGS (**S2B Fig**), indicating that knock-down of *NbDCP1*, *NbDCP2*, *NbXRN4* and *NbPARN* specially inhibits S-PTGS, and expression of unrelated dsRNA does not overload and inhibit the silencing machinery. When 35S-GFP and 35S-dsGF were co-expressed with dsNbDCP1, dsNbDCP2, dsNbXRN4 or dsNbPARN in *N*. *benthamiana*, no GFP fluorescence was detected in the co-infiltrated areas, similar to the Vec-infiltrated leaf patches (**Fig 4D**). In contrast, strong GFP signals were evidenced in the P19 co-expressing patches. GFP-specific siRNA blots showed that in comparison with Vec, silencing of *NbDCP1*, *NbDCP2*, *NbXRN4* or *NbPARN* drastically supressed the production of secondary siRNAs (‘P’ siRNAs) during S-PTGS, but showed no obvious changes in the accumulation of primary siRNAs (‘GF’ siRNAs) during IR-PTGS (**Fig 4E and 4F**). These data support that NbDCP1, NbDCP2, NbXRN4 or NbPARN participate in GFP-induced S-PTGS, but are not involved in IR-PTGS.

### NbDCP1, NbDCP2, NbXRN4 and NbPARN degrade *GFP* RNA via the RNA decay pathway in RDR6-deficient plants

**Fig 5:**
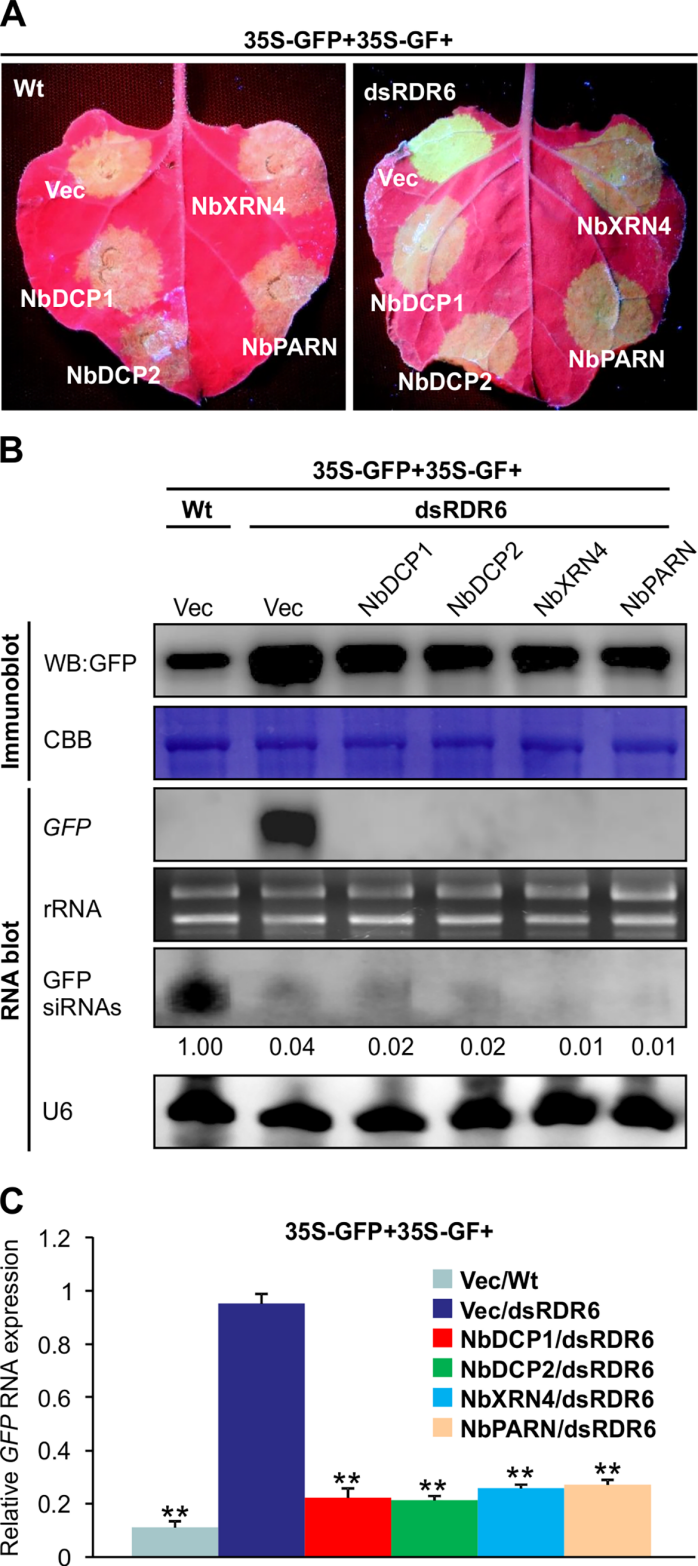
Expression of NbDCP1, NbDCP2, NbXRN4, or NbPARN suppresses *GFP* expression in RDR6-deficient *N*. *benthamiana* plants. (**A**) Visualization of green fluorescence of representative agroinfiltrated leaves. Pictures of representative agroinfiltrated leaves were taken at 5 dpi under UV light. Wild type (Wt) *N*. *benthamiana* or dsRDR6 transgenic *N*. *benthamiana* (dsRDR6) plants were agroinfiltrated with three expression vectors including 35S-GFP, 35S-GF and one of the following vectors: an empty vector (Vec) as a control, Myc-tagged-NbDCP1, NbDCP2, NbXRN4, and NbPARN. Similar results were obtained from three independent experiments. (**B**) Accumulation of GFP protein, *GFP* mRNA and siRNAs in the infiltrated leaves shown in (A) at 5 dpi. CBB staining of the large subunit of Rubisco, ethidium bromide staining of rRNA, and U6 serve as a loading control for immunoblot, mRNA blot and siRNA blot, respectively. (**C**) Relative accumulation of *GFP* mRNAs analyzed by specific qRT-PCR in the infiltrated leaves shown in (A) at 5 dpi. *NbActin* serves as an internal standard. Each mean value was calculated based on three independent experiments (n = 3 samples). Values represent the mean ± SD. Double asterisks indicate a highly significant difference compared to 35S-GFP+35S-GF+Vec/dsRDR6 (*P* < 0.01, Student’s *t* test). The values of GFP siRNAs/U6 were quantified by ImageJ software and then were normalized against the mean value corresponding to the Vec treatment in Wt *N*. *benthamiana* plants, which was set to 1.00.

To analyze the role of NbDCP1, NbDCP2, NbXRN4 and NbPARN in RNA decay in the absence of S-PTGS, we used dsRDR6 transgenic plants, in which sense *GFP* induced S-PTGS is compromised [32]. Three expression vectors including 35S-GFP, 35S-GF and one of the following vectors: NbDCP1, NbDCP2, NbXRN4, NbPARN and an empty vector (Vec) were co-infiltrated into dsRDR6 transgenic *N*. *benthamiana* leaves. At 5 dpi, the leaf patch infiltrated with 35S-GFP, 35S-GF and Vec still showed that strong GFP fluorescence owing to the suppression of S-PTGS in dsRDR6 *N*. *benthamiana* leaves compared to the corresponding patch in wild type (Wt) *N*. *benthamiana* leaves (**Fig 5A**). Expression of NbDCP1, NbDCP2, NbXRN4 or NbPARN in dsRDR6 transgenic plants reduced GFP fluorescence in comparison with the Vec control (**Fig 5A**). qRT-PCR, RNA and protein gel blot analyses revealed that the reduced fluorescence in leaf patches expressing NbDCP1, NbDCP2, NbXRN4 or NbPARN was accompanied by a reduction of both *GFP* mRNA and protein, but without an increase of GFP-specific siRNAs (**Fig 5B and 5C**). These data suggest that in RDR6-deficient plants, NbDCP1, NbDCP2, NbXRN4 or NbPARN suppresses *GFP* expression through the RNA decay pathway. In addition, 35S-GFP and 35S-GF were also co-infiltrated with dsNbDCP1, dsNbDCP2, dsNbXRN4 or dsNbPARN in dsRDR6 leaf patches. Consistent with our data in **Fig 4**, much stronger fluorescence and more GFP RNA accumulations were observed when dsNbDCP1, dsNbDCP2, dsNbXRN4 or dsNbPARN was co-expressed with 35S-GFP and 35S-dsGF compared to Vec at 7 dpi (**S3 Fig**). Taken together, these data suggest that all four 5’RDGs promote RNA silencing to target *GFP* RNA in Wt *N*. *benthamiana* plants, and degrade *GFP* transcripts through RNA decay in the RDR6-deficient plants where S-PTGS is blocked. Thus, RNA silencing compared to RNA decay has the priority to degrade *GFP* RNA.

### Viral infection upregulates the RNA decay pathway

**Fig 6:**
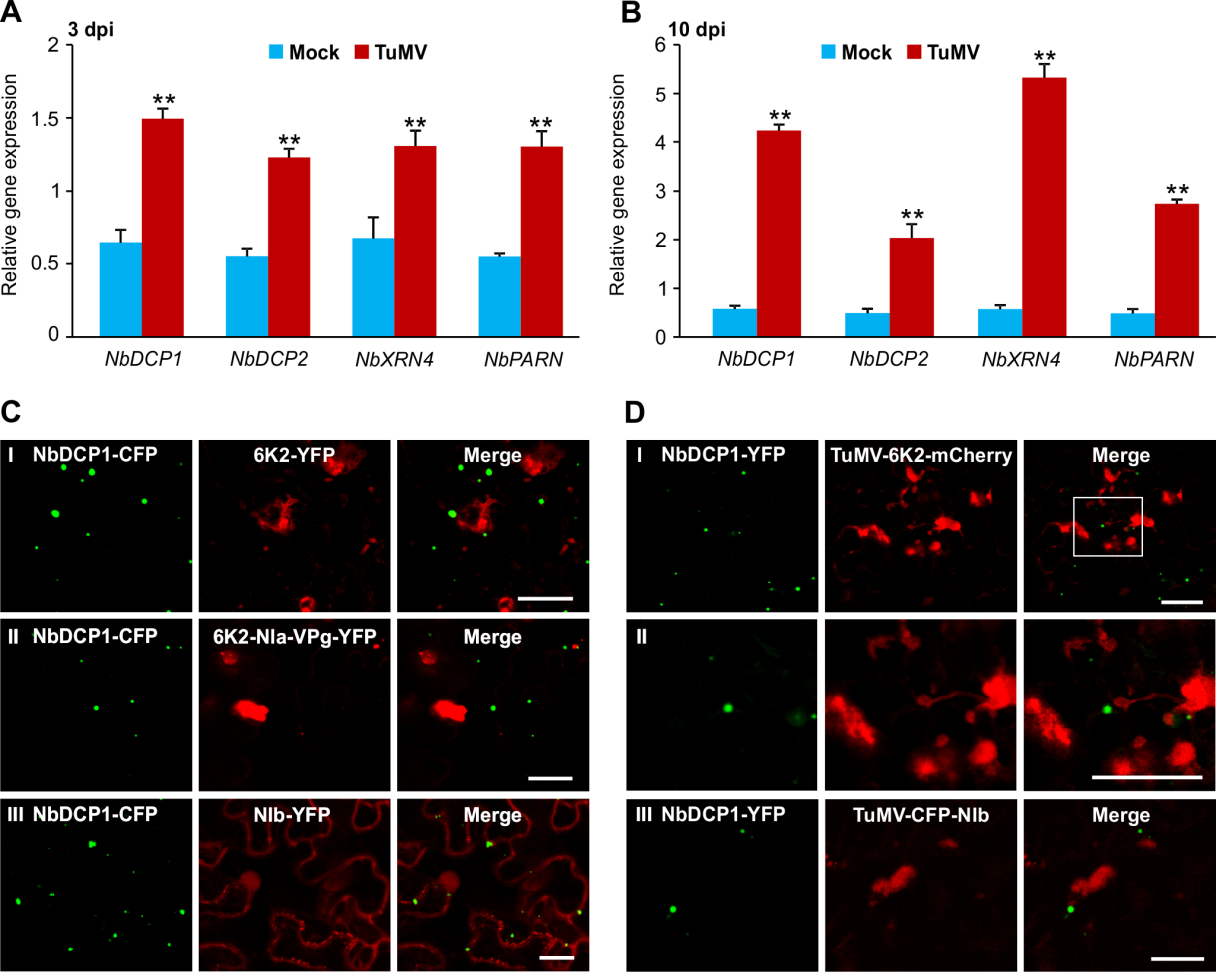
TuMV infection upregulates the RNA decay pathway in *N*. *benthamiana*. **(A, B)** The expression levels of *NbDCP1*, *NbDCP2*, *NbXRN4* and *NbPARN* were analyzed by qRT-PCR in mock (infiltration buffer) or TuMV-infiltrated *N*. *benthamiana* leaves at 3 dpi (A) or upper new leaves at 10 dpi (B). *NbActin* was used as an internal standard. Each mean value was calculated based on three independent biological repeats (n = 3 samples). Values represent the mean ± SD. Double asterisks indicate a highly significant difference compared to mock at 3 dpi (A) or 10 dpi (B) (*P* < 0.01, Student’s *t* test). (**C**) Confocal microscopy analysis of cells co-expressing NbDCP1-CFP (green) and 6K2-YFP (panel I, red), or 6K2-NIa-VPg-YFP (panel II, red), or NIb-YFP (pane III, red) at 32 hpi. Bars, 25 μm. **(D)** Confocal microscopy of cells co-expressing NbDCP1-YFP (green) and TuMV-6K2-mCherry (panel I and panel II, red) or TuMV-CFP-NIb (panel III, red) at 72 hpi. The enlarged image of the area in the white box in panel I is shown in panel II. Bars = 50 μm.

To clarify the role of RNA decay in TuMV infection, we determined the expression levels of the four *5’RDGs* in TuMV-infected local and systemic leaves of *N*. *benthamiana* plants at 3 and 10 dpi by using qRT-PCR (Fig 6A and 6B). The expression levels of all the four *5’RDGs* were significantly upregulated in both local and systemic leaves in response to TuMV infection (**Fig 6A and 6B**). We further checked whether TuMV replication proteins or replication vesicles are associated with P-bodies. The decapping protein DCP1 is a well-established marker for P-bodies in plants [11,33]. We co-expressed NbDCP1-CFP and three TuMV-encoded replication-required proteins including 6K2 (which induces the formation of viral replication vesicles for virus replication), 6K2-NIa-VPg (containing the genome-linked viral protein VPg) and NIb (the only viral RNA-dependent RNA polymerase) with YFP tagged to their respective C-terminus. No typical co-localization signals were observed between NbDCP1 and these viral proteins (**Fig 6C**). Possible co-localization between 5’RDGs and viral replication proteins was also tested using two TuMV infectious clones TuMV-6K2-mCherry and TuMV-CFP-NIb [34,35]. The former contains an extra copy of mCherry-tagged 6K2, and in the latter, NIb is tagged by an N-terminal CFP. No co-localization was found between NbDCP1 and the 6K2-labelled viral replication vesicles or CFP-tagged NIb (**Fig 6D**). These suggest that TuMV infection upregulates the RNA decay pathway which is not apparently associated with the virus replication complex.

### Co-expression of NbDCP1, NbDCP2, NbXRN4 or NbPARN inhibits TuMV RNA accumulation

**Fig 7:**
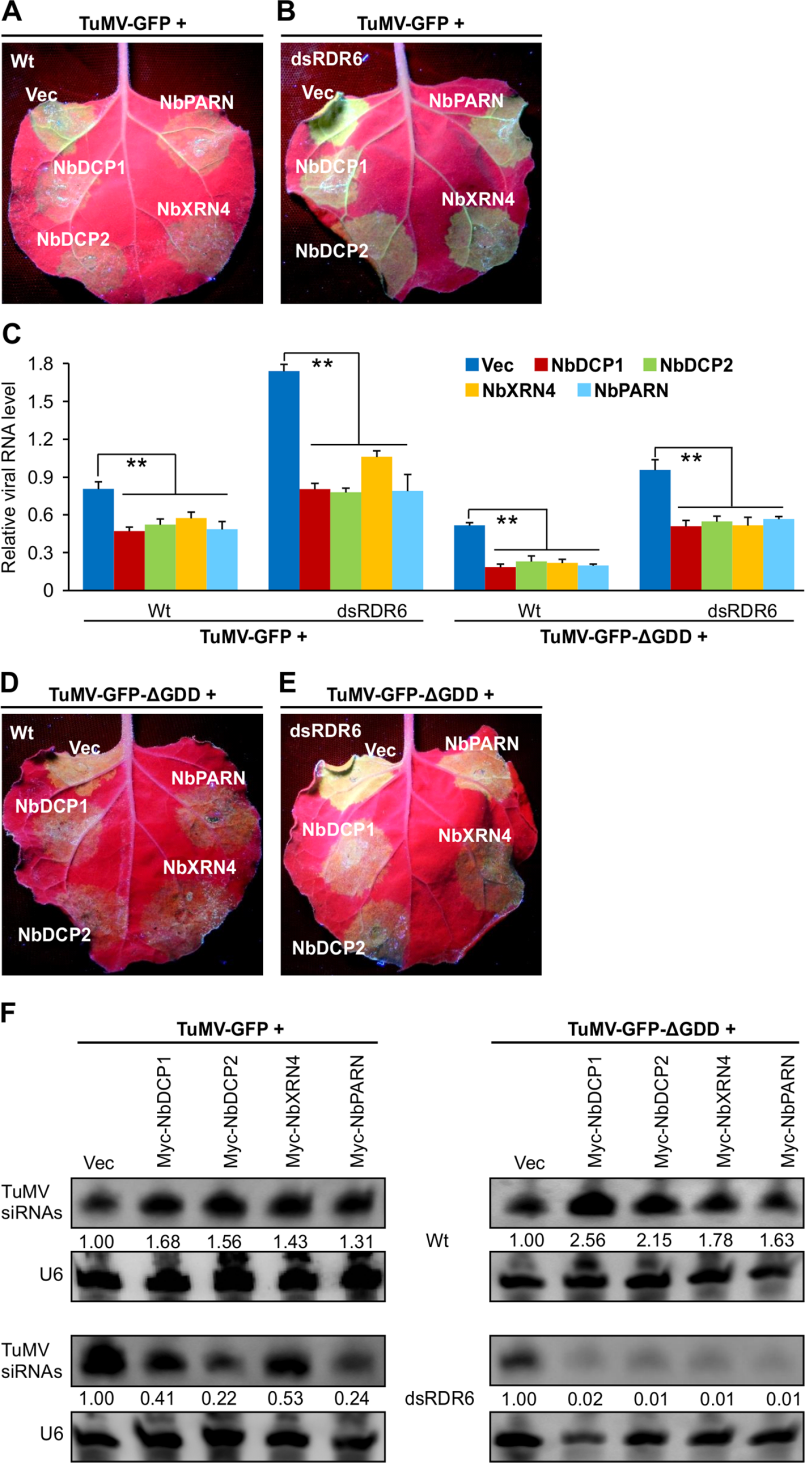
Expression of NbDCP1, NbDCP2, NbXRN4, or NbPARN reduces TuMV RNA accumulation. **(A, B)** GFP fluorescence in Wt or RDR6-deficient (dsRDR6) *N*. *benthamiana* leaves co-infiltrated with TuMV-GFP and one of the following vectors: Vec, NbDCP1, NbDCP2, NbXRN4 or NbPARN at 3 dpi. **(C)** qRT-PCR analyses of TuMV RNA levels. RNA was extracted from the infiltrated patches shown in (A) at 3 dpi. Each value was normalized against *NbActin* transcripts in the same sample. Error bars represent SD (n = 3 independent biological repeats). Double asterisks indicate a highly significant difference compared to the treatment of Vec (*P* < 0.01, Student’s *t* test). **(D, E)** GFP fluorescence in Wt or RDR6-deficient (dsRDR6) *N*. *benthamiana* leaves co-infiltrated with TuMV-GFP-ΔGDD (a replication-defective mutant) and one of the following vectors: Vec, NbDCP1, NbDCP2, NbXRN4 or NbPARN at 3 dpi. (**F**) Accumulation of TuMV siRNAs in the infiltrated patches shown in (A, B, D, E) at 3 dpi. Northern blotting was performed using DIG-labeled DNA probes complementary to the TuMV genome. U6 serves as a loading control for siRNA blot, respectively. The values of GFP siRNAs/U6 were quantified by ImageJ software and then were normalized against the mean value corresponding to the Vec treatment, which was set to 1.00.

To examine whether these *N*. *benthamiana* 5’RDGs affect TuMV RNA accumulation, TuMV-GFP was co-infiltrated into *N*. *benthamiana* leaves with NbDCP1, NbDCP2, NbXRN4, NbPARN or an empty vector (Vec). At 3 dpi, the leaf patches infiltrated with these *N*. *benthamiana* 5’RDGs showed weaker GFP fluorescence compared to the Vec control (**Fig 7A**). As GFP originated from the recombinant virus, its fluorescent intensity could be considered to be an indicator of TuMV replication. qRT-PCR analyses confirmed the reduced viral RNA levels in leaf patches co-expressing NbDCP1, NbDCP2, NbXRN4 or NbPARN (**Fig 7C**). Consistently, this reduction was accompanied with an increase of TuMV-siRNAs (**Fig 7F**). We also checked if NbDCP1, NbDCP2, NbXRN4 or NbPARN affects TuMV infection in dsRDR6 transgenic *N*. *benthamiana* plants (**Fig 7B**). Overexpression of NbDCP1, NbDCP2, NbXRN4 or NbPARN in dsRDR6 transgenic plants inhibited TuMV infection too, evidenced by attenuated GFP fluorescence and reduced levels of viral RNAs (**Fig 7B and 7C**). Interestingly, remarkable amounts of TuMV-derived siRNAs were detected in the Vec-infiltrated control sample of dsRDR6 transgenic plants, possibly due to primary silencing induced by viral dsRNA (viral RNA replicative intermediates) during robust viral replication (**Fig 7F**). These data demonstrate that expression of 5’RDGs inhibits TuMV RNA accumulation in both Wt and dsRDR6 transgenic plants.

To examine 5’RDGs-mediated antiviral defense without dsRNA-mediated primary silencing in Wt and dsRDR6 transgenic *N*. *benthamiana*, we used a replication-deficient TuMV infectious clone, TuMV-GFP-ΔGDD [36]. This clone allows the transcription of full-length viral RNAs in the plant cell and subsequent translation of viral proteins. As the highly conserved GDD motif in NIb is mutated, the clone loses the ability to biosynthesize RNA and to generate viral RNA replicative intermediates. In comparison with the Vec control, expression of NbDCP1, NbDCP2, NbXRN4 or NbPARN in Wt *N*. *benthamiana* suppressed TuMV-GFP-ΔGDD RNA accumulation and remarkably boosted the level of TuMV-siRNAs (**Fig 7F**). In dsRDR6 transgenic plants, overexpression of NbDCP1, NbDCP2, NbXRN4 or NbPARN reduced the accumulation of TuMV-GFP-ΔGDD transcripts and supressed the generation of TuMV-GFP-ΔGDD transcripts-derived siRNAs (**Fig 7F**). These data suggest that NbDCP1, NbDCP2, NbXRN4 or NbPARN may degrade TuMV RNA via the RNA decay pathway when PTGS is disrupted.

To minimize the interference of input RNA, we also conducted this assay with a very low concentration of agrobacterial cultures (OD600 = 0.05) harboring TuMV or TuMV-ΔGDD. As expected, in TuMV-infiltrated Wt or dsRDR6 transgenic plants, viral RNA accumulation was significantly higher than that in the leaves infiltrated with TuMV-ΔGDD (**S4A Fig**). Overexpression of NbDCP1, NbDCP2, NbXRN4 or NbPARN, compared to Vec, reduced TuMV or TuMV-ΔGDD RNA accumulations. Overexpression of these RNA decay components also resulted in an obvious decrease of TuMV-derived siRNAs (**S4B, Fig 7F**). Due to infiltration with a very lower concentration of agrobacterial culture, no detective viral siRNAs were found in TuMV-ΔGDD-infiltrated leaves. These results further suggest that during viral replication, siRNAs mainly derive from viral RNA replicative intermediates, which are proportional to viral RNA accumulations, rather than from RDR6-dependent secondary siRNAs. Taken together, these data support that 5’RDGs act as plant defense against TuMV RNA accumulation.

### Knock-down of *NbDCP1*, *NbDCP2*, *NbXRN4* or *NbPARN* enhances viral local and systemic infections

**Fig 8:**
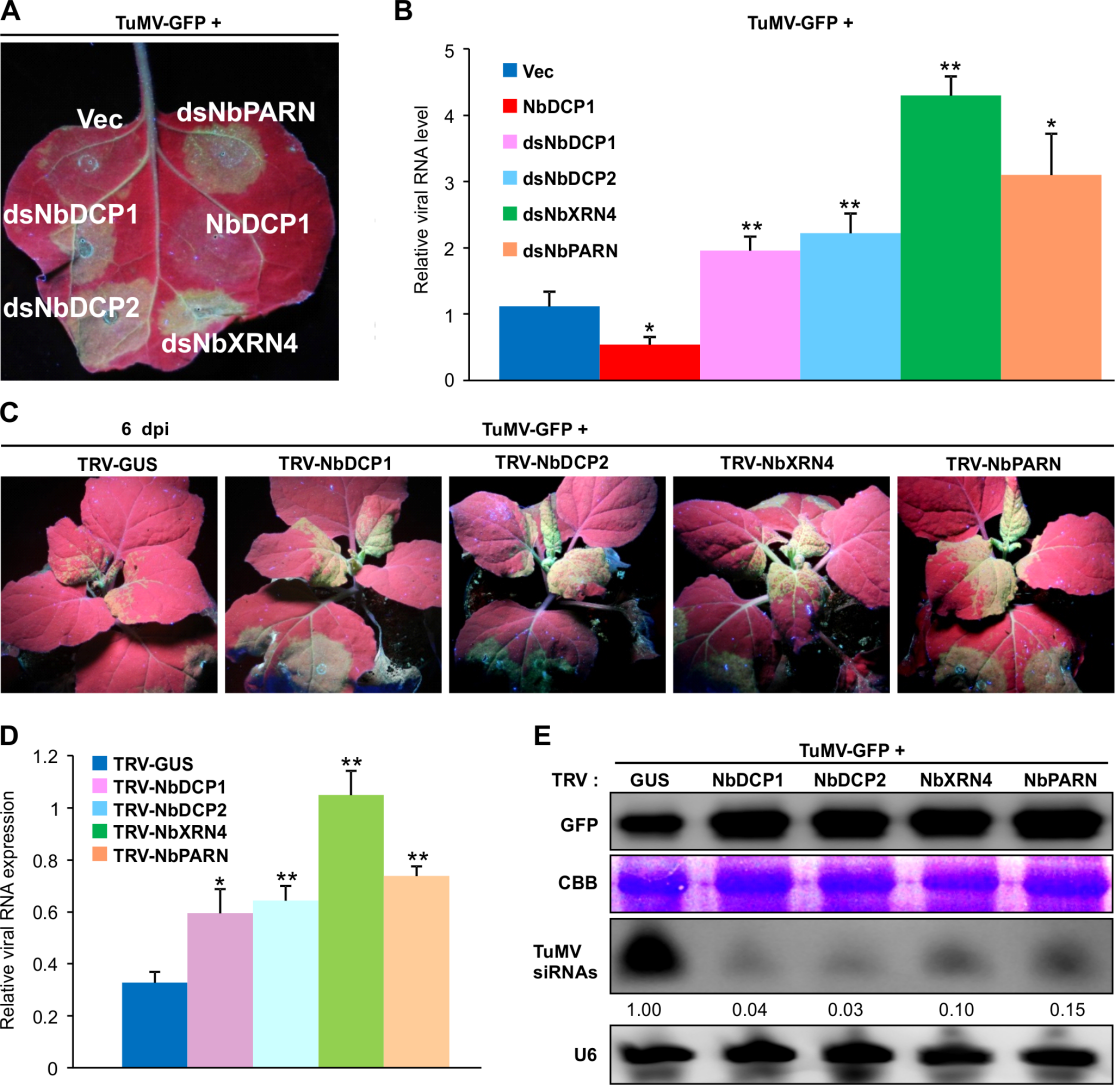
Knock-down of *NbDCP1*, *NbDCP2*, *NbXRN4* or *NbPARN* facilitates TuMV infection. **(A)** GFP fluorescence in *N*. *benthamiana* leaves co-infiltrated with TuMV-GFP and one of the following expression vectors: an empty vector (Vec) as a control, NbDCP1, dsNbDCP1, dsNbDCP2, dsNbXRN4 and dsNbPARN at 4 dpi. **(B)** Relative TuMV RNA levels determined by qRT-PCR. RNA was extracted from the infiltrated patches shown in (A) at 4 dpi. Each value was normalized against *NbActin* transcripts in the same sample. Error bars represent SD (n = 3 independent biological repeats). *, *P* < 0.05; **, *P* < 0.01 (Student’s *t* test). (**C**) GFP fluorescence in systemic leaves of plants pre-treated with TRV-GUS, TRV-NbDCP1, TRV-NbDCP2, TRV-NbXRN4, or TRV-NbPARN and then infected by TuMV-GFP was photographed under UV light at 6 dpi. (**D**) Relative TuMV RNA levels determined by qRT-PCR. RNA was extracted from plants in (C) at 14 dpi. *, *P* < 0.05; **, *P* < 0.01 (Student’s *t* test). (**E**) Accumulation of GFP protein and TuMV siRNAs in the systemic leaves of plants in (C) at 14 dpi. CBB staining of the large subunit of Rubisco and U6 serve as a loading control for immunoblot, mRNA blot and siRNA blot, respectively. The values of GFP siRNAs/U6 were quantified by ImageJ software and then were normalized against the mean value corresponding to the TRV-GUS treatment, which was set to 1.00.

To further investigate the effect of silencing of *5’RDGs* on TuMV infection, *N*. *benthamiana* leaf patches were agroinfiltrated with TuMV-GFP and one of the following vectors: an empty vector (Vec) as a control, NbDCP1, dsNbDCP1, dsNbDCP2, dsNbXRN4 and dsNbPARN. At 4 dpi, knock-down of *NbDCP1*, *NbDCP2*, *NbXRN4* or *NbPARN* led to the increased levels of TuMV-GFP fluorescence, GFP proteins, and TuMV RNA, and a reduced level of TuMV siRNAs (**Fig 8A and 8B and S5 Fig**). Thus, silencing of *NbDCP1*, *NbDCP2*, *NbXRN4* or *NbPARN* promoted TuMV RNA accumulations possibly through suppression of TuMV-derived siRNA production. To verify if this also holds true for TuMV systemic infection, a modified TRV VIGS vector carrying the partial sequence of GUS (as a control), *NbDCP1*, *NbDCP2*, *NbXRN4* or *NbPARN* was pre-inoculated into *N*. *benthamiana* to knock down *NbDCP1*, *NbDCP2*, N*bXRN4* or *NbPARN* expression (**S6 Fig**). Silenced plants were then inoculated with TuMV-GFP. At 6 dpi, GFP signals started to appear along the veins in newly developed leaves under UV lamp in TRV-GUS-treated plants (**Fig 8C**). However, all *NbDCP1*, *NbDCP2*, *NbXRN4* or *NbPARN*-silenced plants developed much stronger GFP signals in the corresponding leaves (**Fig 8C**). Consistently, higher levels of viral RNAs and GFP proteins were found in the *5’RDGs*-silenced plants (**Fig 8D and 8E**). It was also evidenced that silencing of *5’RDGs* inhibited TuMV-derived siRNAs (**Fig 8E)**. These data suggest that knock-down of *5’RDGs* facilitates TuMV infection and suppresses viral siRNAs in *N*. *benthamiana*.

To further examine if these 5’RDGs also inhbit TuMV infection in Arabidopsis, we obtained Arabidopsis knockdown mutants of *DCP2* and *PARN*, and knockout mutants of *DCP1* and *XRN4* (**S7 Fig**). In comparison with Wt Arabidopsis plants, all mutants showed enhanced susceptibility to TuMV infection by accumulation of higher levels of viral RNA (**S8 Fig**). Clearly, 5’RDGs play an anti-TuMV role in both *N*. *benthamiana* and Arabidopsis species.

### VPg interacts with and targets DCP2 to the nucleus to inhibit the formation of the cytoplasmic DCP1/DCP2 granules

**Fig 9:**
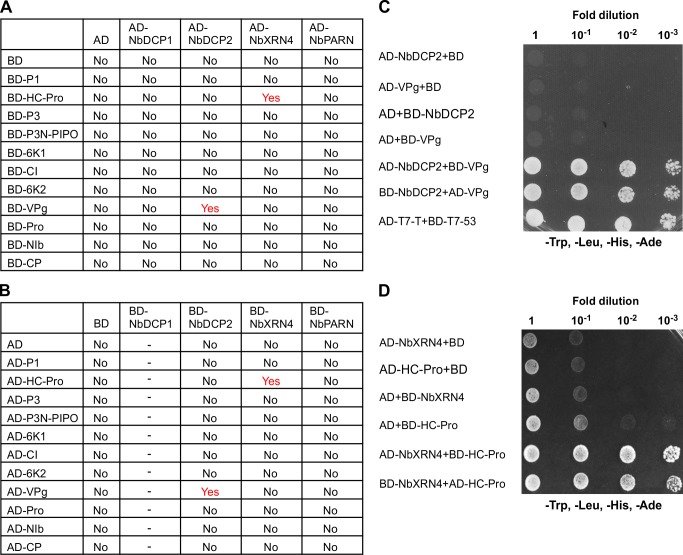
Yeast two-hybrid (Y2H) assays between the four 5’RDGs and 11 TuMV viral proteins. (**A, B**) Summary of Y2H assay results. Y2H Gold yeast strains co-transformed with the indicated plasmids were plated on synthetic dextrose (SD)/-Trp, -Leu, -His, -Ade medium to identify protein interactions at 3 days after transformation. Proteins were fused to either the Gal4 DNA binding (BD) or activation (AD) domain. No means no positive interaction between two tested proteins, Yes means positive interaction between two tested proteins, and ‘-’ means that NbDCP1 has self-activation activity when fused to BD vector, indicating a unauthentic interaction. **(C, D)** Y2H assays for NbDCP2 and VPg (C), and NbXRN4 and HC-Pro (D). Y2H Gold yeast strains co-transformed with the indicated plasmids were subjected to 10-fold serial dilutions and plated on SD/-Trp, -Leu, -His, -Ade medium to identify protein interactions at 3 days after transformation. Cells co-transformed with AD-T7-T+BD-T7-53 serve as positive controls; cells co-transformed with AD-NbDCP2 or AD-NbXRN4 and the empty BD, or the empty AD and BD-NbDCP2 or BD -NbXRN4 are negative controls.

To screen for possible protein-protein interactions between the four 5’RDGs and 11 TuMV proteins, we conducted yeast two-hybrid (Y2H) assays. All the tested proteins were fused with the GAL4 transcription activation domain (AD) and the GAL4 DNA binding domain (BD). As summarized in **Fig 9A and 9B**, regardless of whether AD or BD fusions were used for the assay, positive interactions were only found between NbDCP2 and VPg, and between NbXRN4 and HC-Pro.

**Fig 10:**
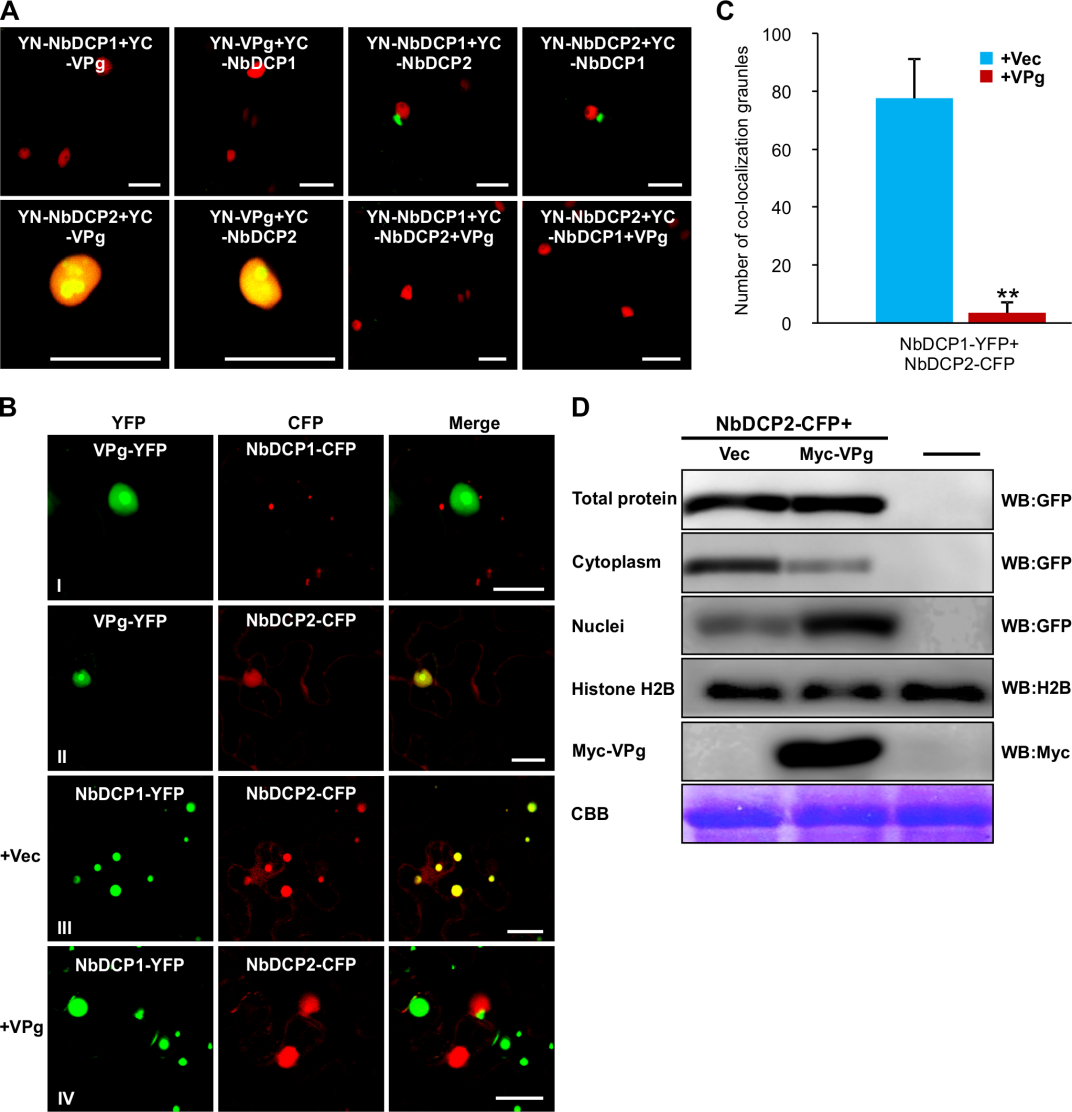
VPg negatively regulates the formation of cytoplasmic NbDCP1/NbDCP2 granules. **(A)** BiFC assays for possible protein-protein interactions *in planta*. NbDCP1, NbDCP2 and VPg were fused with YN or YC. The fused proteins were transiently expressed in H2B-RFP transgenic *N*. *benthamiana* leaves. Confocal microscopy was carried out at 32 hpi. Yellow fluorescence (green) was observed in the leaf cells co-expressing NbDCP1 + NbDCP2 or NbDCP2 + VPg, but not in the cells co-expressing NbDCP1 + VPg, or NbDCP1 + NbDCP2 in the presence of VPg. Nuclei of tobacco leaf epidermal cells are indicated by expression of the H2B-RFP transgene (red). Bars = 25 μm. **(B)** Co-localization of VPg + NbDCP1, VPg + NbDCP2, NbDCP1 + NbDCP2 in the presence of an empty vector (+Vec) or VPg (+VPg) in wild type *N*. *benthamiana* leaf cells. Confocal microscopy was carried out at 32 hpi. Bars = 25 μm. **(C)** The average number of NbDCP1/NbDCP2 co-localization granules (per 10 cells) when they were co-infiltrated with Vec (+Vec) or VPg (+VPg). Independent infiltration experiments were repeated three times and 30 cells in total were used to quantify. Values represent the mean number of the NbDCP1/NbDCP2 granules (per 10 cells) ± SD. Student’s *t* test was performed to compare differences, and double asterisks indicate a highly significant difference (*P* < 0.01). (**D**) Immunoblotting analyses of NbDCP2-CFP at 2 dpi. Total proteins or proteins isolated from the cytoplasm or nuclei were probed with GFP antibodies, and total proteins were also incubated with Myc antibodies to detect Myc-VPg. Equal loading for the nuclear and total protein samples was monitored by probing with Histone H2B antibodies and CBB staining, respectively.

The interaction between NbDCP2 and VPg was further confirmed by BiFC in transgenic *N*. *benthamiana* expressing H2B-RFP as a nuclear marker (**Fig 10A**). The NbDCP2 and VPg interaction was detected in the nucleus (**Fig 10A and S10 Fig**). Consistently, NbDCP2 interacted with NbDCP1 (serving as a positive control) and formed bright granules in the cytoplasm, and no interaction was found between NbDCP1 and VPg (**Fig 10A**). The fact that NbDCP2 interacts with VPg in the nucleus, and with NbDCP1 in the cytoplasm to form the decapping complex that is required for 5’–3’ RNA decay [11,33] prompted us to investigate whether TuMV VPg negatively regulates the assembly of the NbDCP1/NbDCP2 decapping complex. To test this hypothesis, YN-NbDCP1 and YC-NbDCP2 or YN-NbDCP2 and YC-NbDCP1 were co-infiltrated with VPg into the H2B-RFP leaves. No interaction signals between NbDCP1 and NbDCP2 were detected when VPg was co-expressed (**Fig 10A**), suggesting that VPg indeed disrupts the NbDCP1 and NbDCP2 interaction.

We further observed the subcellular co-localization of VPg, NbDCP1 and NbDCP2. *N*. *benthamiana* leaves were co-infiltrated to transiently co-express VPg-YFP and NbDCP1-CFP or NbDCP2-CFP. No co-localization was observed for VPg-YFP and NbDCP1-CFP, whereas VPg-YFP co-localized with NbDCP2-CFP in the nucleus (**Fig 10B,** two top rows). Moreover, co-expression of VPg remarkably suppressed the formation of the NbDCP1/NbDCP2 granules in the cytoplasm, in comparison with the vector control (**Fig 10B**, two bottom rows, **and 10C**). Initially, we speculated that the reduced NbDCP1/NbDCP2 interaction was owing to the reduced NbDCP2 protein level induced by VPg. To test this possibility, total proteins were extracted from the leaves co-infiltrated with NbDCP2-CFP and empty vector (Vec) or Myc-VPg, and Western blot analysis was performed to determine the accumulation of NbDCP2-CFP. Co-expression of Myc-VPg did not obviously affect the NbDCP2-CFP protein level (**Fig 10D**). Subsequently, we considered the possibility that the VPg-NbDCP2 interaction might negatively regulate the distribution of NbDCP2 in the cytoplasm to inhibit the formation of NbDCP1/NbDCP2 cytoplasmic granules. To test this assumption, we extracted the cytoplasmic and nuclear proteins separately, and performed Western blot analysis to determine the accumulation of NbDCP2-CFP in the cytoplasm and nuclear. Indeed, when Myc-VPg was co-expressed, the amount of NbDCP2-CFP obviously decreased in the cytoplasm but remarkably increased in the nucleus (**Fig 10D**). These data suggest that TuMV VPg interferes with the interaction between NbDCP1 and NbDCP2 by targeting NbDCP2 from the cytoplasm to nucleus.

### HC-Pro interacts with XRN4 and supresses XRN4 activity

**Fig 11:**
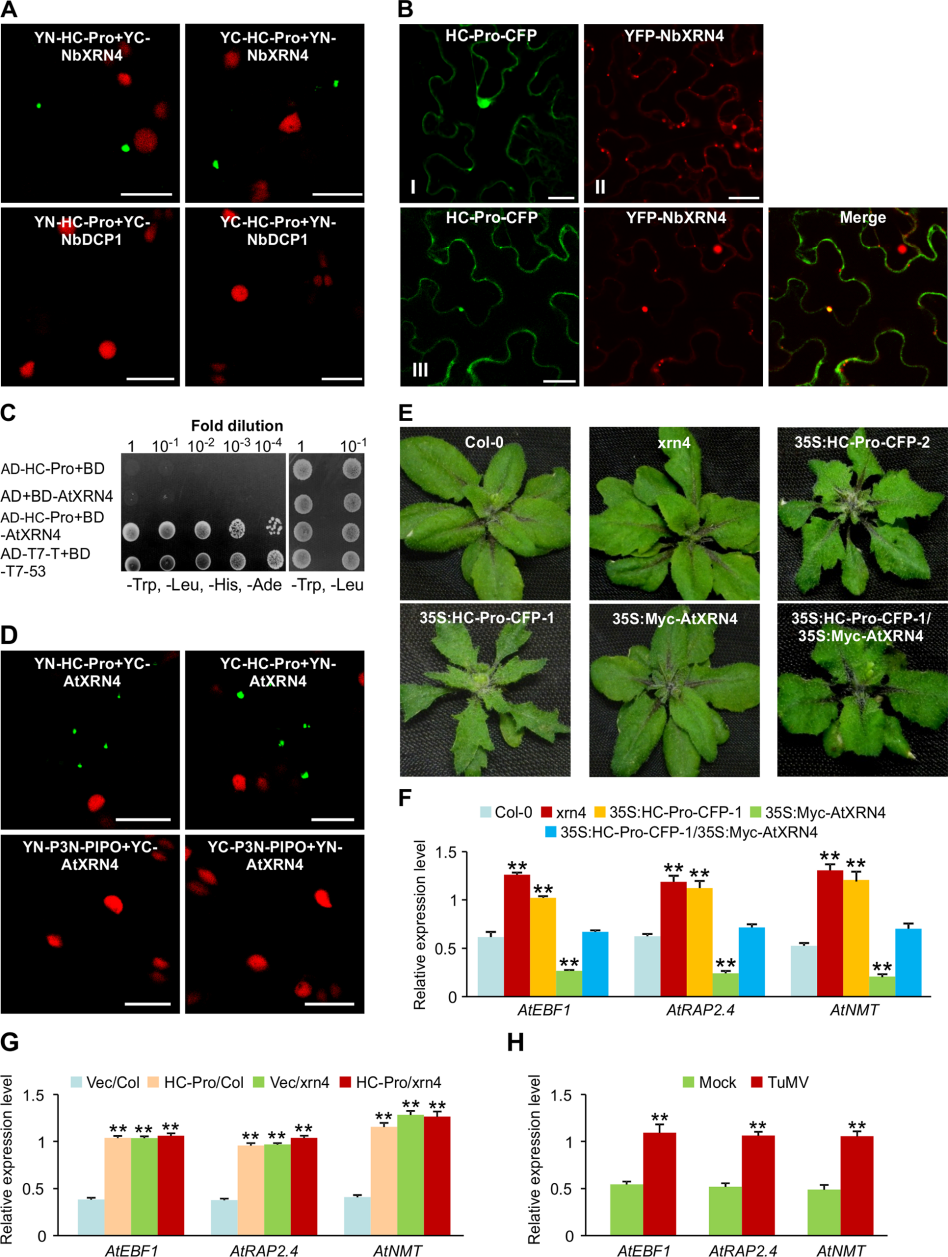
HC-Pro interacts with XRN4 and inhibits XRN4 activity. **(A)** BiFC assays to confirm the HC-Pro and NbXRN4 interaction in H2B-RFP *N*. *benthamiana* leaves at 32 hpi. HC-Pro and NbXRN4 were fused with YN and YC. Yellow fluorescence (green) was observed in the cells co-expressing YN-HC-Pro+YC-NbXRN4 or YC-HC-Pro+YN-NbXRN4. Co-expression of YN-HC-Pro+YC-NbDCP1 or YC-HC-Pro+YN-NbDCP1 did not result in any detectable fluorescence in H2B-RFP *N*. *benthamiana* leaves at 32 hpi, which revealed that HC-Pro did not interact with NbDCP1. Bars = 25 μm. **(B)** Co-localization analysis of HC-Pro-CFP and YFP-NbXRN4 in *N*. *benthamiana* leaves at 32 hpi. Panel I: HC-Pro-CFP was expressed alone, Panel II: YFP-NbXRN4 was expressed alone, Panel III: HC-Pro-CFP and YFP-NbXRN4 were expressed together. Bars = 25 μm. (**C**) Y2H assays for AtXRN4 and HC-Pro. Y2H Gold yeast strains co-transformed with the indicated plasmids were subjected to 10-fold serial dilutions and plated on SD/-Trp, -Leu, -His, -Ade medium to identify protein interactions at 3 days after transformation. Cells co-transformed with AD-T7-T+BD-T7-53 serve as positive controls; cells co-transformed with AD-HC-Pro and the empty BD, or the empty AD and BD-AtXRN4 are negative controls. (**D**) BiFC assays revealed that HC-Pro interacted with AtXRN4 in H2B-RFP *N*. *benthamiana* leaves at 32 hpi. P3N-PIPO and AtXRN4 served as a negative control for the protein-protein interaction assay. Bars = 25μm. **(E)** Phenotypes of Col-0, *xrn4* mutant, and transgenic Arabidopsis plants transformed with 35S:Myc-AtXRN4, 35S:HC-Pro-CFP-1, 35S:HC-Pro-CFP-2 and 35S:HC-Pro-CFP-1/35S:Myc-AtXRN4 at 20 days after sowing. 35S:HC-Pro-CFP-1/35S:Myc-AtXRN4 plants were obtained by genetic crosses between 35S:HC-Pro-CFP-1 and 35S:Myc-AtXRN4 Arabidopsis plants. T2 generation plants were used in the above experiments. The confirmation of *xrn4* mutant, transgenic plants expressing 35S:Myc-AtXRN4, 35S:HC-Pro-CFP-1, 35S:HC-Pro-CFP-2 and 35S:HC-Pro-CFP-1/35S:Myc-AtXRN4 was shown in **S7 Fig** and **S10 Fig**. (**F, G, H**) qRT-PCR analysis of *AtEBF1*, *AtRAP2*.*4*, *AtNMT* expression in Col-0, *xrn4* mutant, and transgenic plants carrying 35S:Myc-AtXRN4, 35S:HC-Pro-CFP-1 and 35S:HC-Pro-CFP-1/35S:Myc-AtXRN4 at 20 days after sowing (F), in Col-0 and *xrn4* mutant leaves infiltrated by empty vector (Vec) and HC-Pro at 3 dpi (G), and in Col-0 newly emerged leaves of mock- or TuMV-infected plants 12 dpi (H). *AtActinII* gene was used as an internal control. The data were analyzed using Student’s *t* test and asterisks denote significant differences between treatments (* *P* <0.05, ** *P* <0.01).

The interaction between HC-Pro and NbXRN4 was verified by BiFC assays in *N*. *benthamiana*. Expression vectors YN-HC-Pro, YC-HC-Pro, YN-NbXRN4 and YC-NbXRN4 were generated to express HC-Pro or NbXRN4 fusions with YN or YC, respectively. Pairwise expression of YN-HC-Pro and YC-NbXRN4 or YN-NbXRN4 and YC-HC-Pro by agroinfiltration resulted in strong YFP fluorescence signals in the cytoplasm at 32 hpi (**Fig 11A**). No YFP fluorescence was detectable in the leaf sample co-expressing HC-Pro and NbDCP1, which serves as a negative control (**Fig 11A**). We also examined the subcellular localization of HC-Pro and NbXRN4 in *N*. *benthamiana* leaf epidermal cells co-expressing CFP-tagged HC-Pro (HC-Pro-CFP) and YFP-tagged NbXRN4 (YFP-NbXRN4). We found that HC-Pro-CFP localized in the cytoplasm and nucleus, and some formed granules in the cytoplasm (**Fig 11B, panel I**). HC-Pro-CFP co-localized with NbXRN4-CFP to form a bright dot in the cytoplasm (**Fig 11B, panel III**). These results confirm that TuMV HC-Pro interacts with NbXRN4.

To assess the biological relevance of the HC-Pro and XRN4 interaction, we used the model plant *Arabidopsis*. At first, we confirmed that TuMV HC-Pro indeed interacts with AtXRN4, the ortholog of NbXRN4 in Arabidopsis by conducting Y2H assays in yeast (**Fig 11C**) and BiFC assays in *N*. *benthamiana* (**Fig 11D**). Then, we obtained an Arabidopsis *xrn4* mutant and generated transgenic Arabidopsis plants expressing HC-Pro and AtXRN4. Interestingly, the phenotype of the *xrn4* mutant mimicked the mild phenotype of HC-Pro transgenic Arabidopsis plants (**Fig 11E**), which displayed serrated leaf edges. Since the P-body is the RNA decay site, and HC-Pro interacts with XRN4 in the cytoplasmic granules resembling the P-body (**Fig 11A and 11D**), we speculated that expression of HC-Pro might suppress AtXRN4 function in RNA decay. To test this idea, three potential substrates of AtXRN4 in RNA decay including *AtEBF1*, *AtRAP2*.*4* and *AtNMT* [37] were analyzed. We found that the mRNA levels of these three genes indeed significantly increased in the *xrn4* mutant Arabidopsis plants and decreased in the XRN4 overexpression transgenic plants (**Fig 11F**). Overexpression of HC-Pro significantly enhanced the mRNA accumulation of *AtEBF1*, *AtRAP2*.*4* and *AtNMT*, which was antagonized by co-overexpression of AtXRN4 (**Fig 11F**). Consistently, co-overexpression of AtXRN4 partially remedied the typical phenotype induced by overexpression of HC-Pro (**Fig 11E**) and the expression levels of *AtEBF1*, *AtRAP2*.*4* and *AtNMT* were similar in HC-Pro-expressing Wt (Col-0) and *xrn4* mutant plants (**Fig 11G**). Moreover, in TuMV-infected plants, the level of these XRN4 substrates was elevated (**Fig 11H**), possibly due to HC-Pro. Taken together, these data suggest that HC-Pro interacts with XRN4 and the interaction inhibits XRN4 activity.

## Discussion

In this study, we found that overexpression of any of four essential RNA decay components 5’RDGs including DCP1, DCP2, XRN4 and PARN failed to suppress GFP-induced S-PTGS and production of siRNAs, and instead enhanced S-PTGS in *N*. *benthamiana* (**Fig 3)**. We also found that knock-down of any of the four *5’RDGs* genes repressed GFP-induced S-PTGS and inhibited the generation of GFP-derived siRNAs (**Fig 4**). These data suggest that 5’RDGs seem to play an additive role to S-PTGS in *N*. *benthamiana*. Our data are consistent with a recent report that the poly(A) tail of mRNA blocks RDR6 from converting canonical mRNAs into substrates for gene silencing and AtRDR6 has an intrinsic preference for poly(A)-less mRNAs over polyadenylated mRNAs as templates in Arabidopsis [38]. However, several previous studies have concluded that both 5′–3′ and 3′–5′ cytoplasmic RNA decay pathways repress S-PTGS in Arabidopsis [28–31]. For example, it has been shown that impairing deadenylation and decapping enhance S-PTGS in Arabidopsis [30,39], possibly through restriction of RNA substrates from entry into the PTGS pathway. However, how deadenylation and decapping blocks the RNA substrate to access to PTGS is yet to be understood. It has also been suggested that RNA decay may compete for the same RNA substrates with RDRs-dependent RNA silencing to supress S-PTGS [28–30]. The assumption was based on the experimental evidence that the deficiency of RNA decay ribonucleases such as XRN4 enhances S-PTGS in Arabidopsis [28–30]. AtXRN4 does not play a significant role in controlling the degradation of unstable transcripts in *A*. *thaliana*, and it degrades predominantly the 5’ uncapped mRNA intermediates as well 3’ mRNA intermediates resulting from miRNA and possibly siRNA-mediated cleavage [37,40]. Moreover, XRN4-mediated decay also preferentially targets some transcripts such as those encoding nucleic acid–binding proteins and chloroplast proteins [40]. We speculate that rather than being competitive for substrates in Arabidopsis, NbXRN4 in *N*. *benthamiana* may degrade RNAs incompletely, generating RNA fragments, which facilitate RDRs-dependent dsRNA synthesis. Clearly, the interplay between RNA decay and RNA silencing is very complicated. The finding from this study may represent another example that not all findings from Arabidopsis can be simply extrapolated to other plant species such as *N*. *benthamiana*. The molecular mechanism by which the RNA decay pathway functions differently in relation to S-PTGS in Arabidopsis and *N*. *benthamiana* needs further study.

RDR6 is required for S-PTGS by conversion of single stranded RNA into dsRNA, and GFP-induced S-PTGS is compromised in RDR6-defective *N*. *benthamiana* plants [32]. In this study, we found that the 5’RDGs suppressed *GFP* expression in dsRDR6 plants, and this was not concomitant with an increment of *GFP* siRNAs (**Fig 5**). Therefore, cytoplasmic RNA decay pathways are involved in the deadenylated or/ and decapped *GFP* RNA degradation. It is well known that silencing of *RDR6* enhances transgene expression such as *GFP* [32,41]. In this study, knock-down of *5’RDGs* in RDR6-defective plants further improves *GFP* expression (**S3 Fig**), suggesting that RNA silencing and RNA decay may coordinate against the over-accumulation of exogenous transcripts. Taken together these data demonstrate that 5’RDGs from *N*. *benthamiana* may process *GFP* RNA to facilitate them into RNA silencing for degradation, and may directly degrade *GFP* RNA via the RNA decay pathway when S-PTGS pathway is interrupted. Thus, RNA silencing compared to RNA decay seems to play a predominant role in the degradation of *GFP* RNA, while both of them constitute an important defense against exogenous RNA.

P bodies are the RNA decay sites, which have been implicated in infection by a number of RNA viruses [20–22,42]. In this study, we found that TuMV infection significantly upregulated the four *5’RDGs* in both the inoculated leaves, and systemically infected leaves of *N*. *benthamiana* plants (**Fig 6A and 6B**). TuMV replication-related proteins or TuMV replication complex were not co-localized with P bodies (**Fig 6C and 6D**), suggesting that these 5’RDGs probably have no access to the virus replication site.

As briefly discussed in Introduction, the cytoplasmic XRN4 inhibits infections by several RNA viruses in *N*. *benthamiana* such as TMV, TBSV, CNV and rice stripe virus infection [15–17,19]. We thus checked if these 5’RDGs affect TuMV RNA accumulation. Consistent with these reports and its roles in *GFP* RNA accumulation, overexpression of the four 5”RDGs including XRN4 supressed TuMV RNA accumulation and silencing of *XRN4* and the other three *5’RDGs* genes promoted TuMV infection in *N*. *benthamiana* (**Fig 7, Fig 8 and S4 Fig**). In addition, we also found that the orthologs of these 5’RDGs in Arabidopsis had the similar antiviral function (**S8 Fig**). Therefore, we conclude that RNA decay is an antiviral pathway to TuMV in both Arabidopsis and *N*. *benthamiana*.

To understand if and how TuMV counteracts the RNA decay machinery for viral infection, we screened possible interactions between 5’RDGs and TuMV encoding proteins. We found that NbDCP2 interacted with VPg, and NbXRN4 interacted with HC-Pro (**Fig 9**). Moreover, we found that the VPg/NbDCP2 interaction disrupted the formation of the NbDCP1/NbDCP2 complex possibly through targeting the cytoplasmic NbDCP2 to the nucleus (**Fig 10 and S5 Fig**). Since the DCP1/DCP2 complex is essential for RNA decay, the VPg/NbDCP2 interaction would affect RNA decay-mediated TuMV RNA degradation. It is worth to mention that VPg is the virus-encoded protein that is covalently linked to the 5' end of the viral genome. Therefore, targeting DCP2 to the nucleus may inhibit the interaction of DCP2 with the genome-linked VPg in the cytoplasm, which can further supresses viral RNA degradation by RNA decay.

Transgenic Arabidopsis plants expressing P1/HC-Pro, a potyvirus-encoded silencing suppressor, causes severe developmental anomalies such as stunting and serrated leaf edges. Initially, defects in both siRNA and microRNA (miRNA) pathways were through to account for the phenotype [43–45]. Subsequent studies showed that ectopic DCL1 largely alleviates developmental anomalies in P1/HC-Pro plants but cannot correct the P1/HC-Pro–associated defects in small RNA pathways, suggesting it is the aberrant Dicer activity that is responsible for developmental defects in the P1/HC-Pro plants [46]. In this study, we found that HC-Pro interacted with NbXRN4 and AtXRN4 in yeast cells and in plant (**Fig 9 and Fig 11**). Arabidopsis *xrn4* mutant plants displayed serrated leaf edges, a developmental defect similar to HC-Pro transgenic plants, and overexpression of AtXRN4 could restore developmental defects in the HC-Pro plants (**Fig 11**), suggesting that the HC-Pro/AtXRN4 interaction may also contribute to the developmental defects through disruption of AtXRN4 activity to host gene expression. This assumption was further supported by the observation that overexpression of HC-Pro increased the level of XRN4 substrates likely via inhibition of XRN4 activity, mimicking the *xrn4* mutant, and by the finding that ectopic overexpression of AtXRN4 mitigated the HC-Pro-induced phenotype and decreased the level of XRN4 substrates (**Fig 11**). Taken these data together, HC-Pro, as a highly efficient VSR of potyviruses, not only suppresses the PTGS and miRNA pathways, but also interferes with the XRN4-mediated 5’–3’ RNA decay pathway.

**Fig 12:**
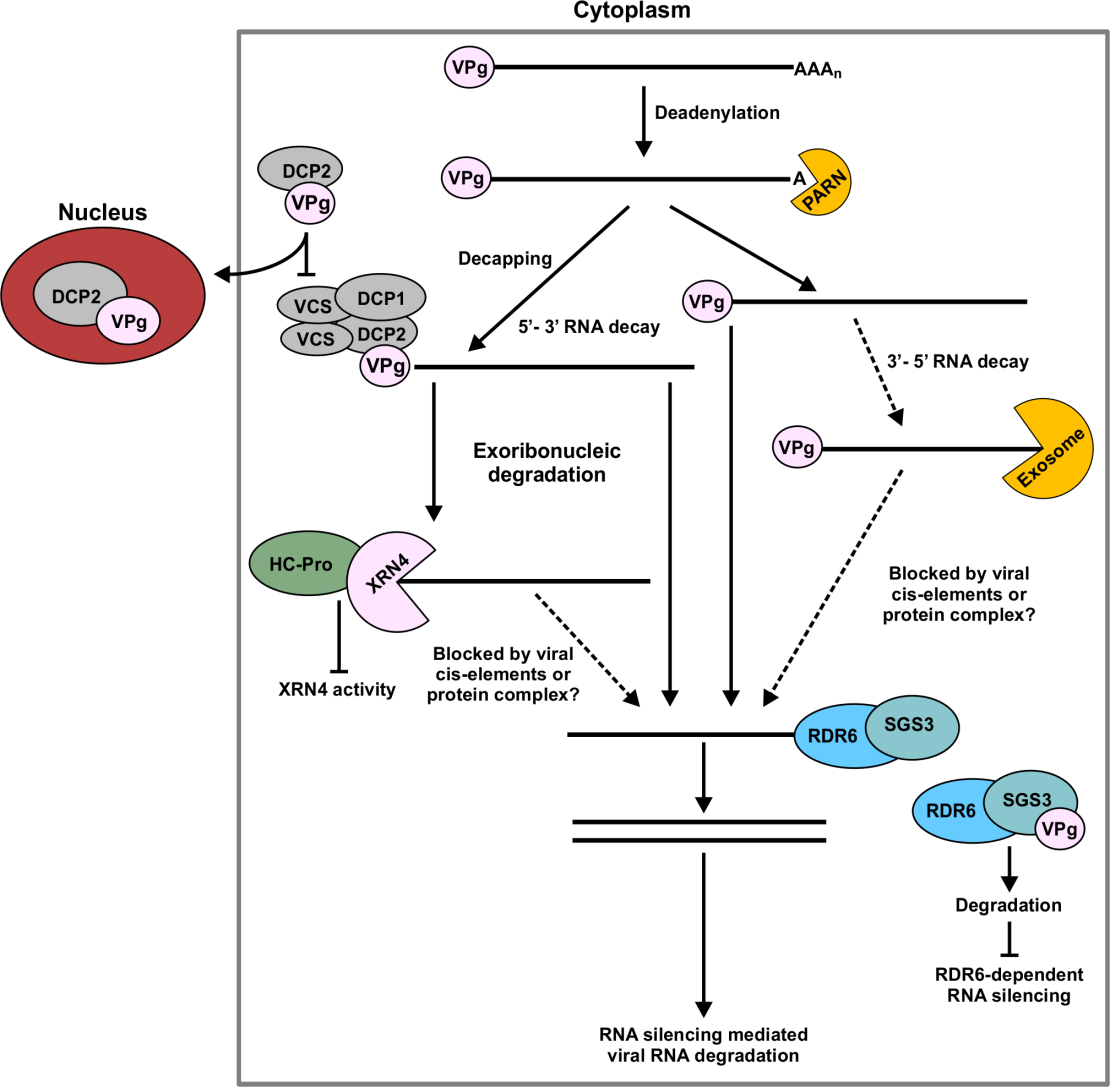
Model for counter-defense by VSRs against cytoplasmic RNA decay- and RNA silencing-mediated plant defenses. After transcription, mRNAs are deadenylated, decapped and degraded by XRN4-mediated 5’–3’ decay or exosome-mediated 3’–5’ degradation. Deadenylated, decapped, or XRN4-partially degraded RNAs facilitate RDR6 to transform ssRNA into dsRNA to degrade the left RNAs by PTGS pathway. Potyviruses encode at least two VSRs: HC-Pro and VPg. HC-Pro interacts with XRN4 to inhibit its slicing activity and VPg disrupts the interaction between NbDCP1 and NbDCP2 by targeting NbDCP2 to the nucleus. As a result, RNA decay-mediated plant defense is compromised. In addition, VPg interacts with SGS3 and mediates its degradation via ubiquitination and autophagy pathways to block RDR6-mediated anti-viral response [36].

Based on the above discussion, we propose a model that in addition to RNA silencing, the RNA decay pathway is another important component of antiviral immunity and viral VSRs function to counteract RNA decay (**Fig 12**). In brief, TuMV genomic RNA is deadenylated, decapped (interfering with or removing VPg) and degraded by XRN4-mediated 5’–3’ decay or exosome-mediated 3’–5’ degradation. Deadenylated, decapped, or XRN4-partially degraded RNAs facilitate RDR6 to transform ssRNA into dsRNA that triggers the PTGS pathway. Typical potyviruses encode two known VSRs: HC-Pro and VPg. HC-Pro interacts with XRN4 to inhibit its slicing activity. Here, HC-Pro as a major virus-encoded suppressor of RNA silencing is not included for discussion, which has been discussed in several reviews [47–49]. VPg may supress RNA decay-mediated degradation of viral RNA through targeting NbDCP2 to the nucleus to disrupt formation of the cytoplasmic NbDCP1/NbDCP2 complex. In addition, VPg interacts with SGS3 and mediates the degradation of SGS3 and its intimidate partner RDR6 via ubiquitination and autophagy pathways to block RDR6-mediated anti-viral response [36].

## Materials and methods

### Plant materials and growth conditions

*N*. *benthamiana* plants were maintained in an insect-free growth chamber at 25 ^o^C and 60% relative humidity under a 16 h light/8 h dark photoperiod. Transgenic dsRDR6 and H2B-RFP lines were kindly provided by David C. Baulcombe (Cambridge University, UK), and Michael M. Goodin (University of Kentucky, USA), respectively. Arabidopsis plants were grown under the similar conditions described above for *N*. *benthamiana*. The Arabidopsis mutants: *dcp1* (SALK_014408C), *dcp2* (SALK_00519), *xrn4* (SALK_014209) and *parn* (Salk_072627) were obtained from the Arabidopsis Biological Resource Center (ABRC) at the Ohio State University. All mutants were confirmed by PCR essentially as previously described [36]. AtXRN4 and HC-Pro transgenic Arabidopsis plants were generated by the floral-dip method [50] and screened by directly spraying of 50 mg/L Basta solutions and subsequently by genomic PCR and DNA sequencing. Western blot and qRT-PCR were used to further confirm transgene expression. 35S:HC-Pro-CFP-1/35S:Myc-AtXRN4 transgenic plants were obtained by genetic crosses between 35S:HC-Pro-CFP-1 and 35S:Myc-AtXRN4-1 Arabidopsis plants and genotyping was done by PCR, qRT-PCR and Western blot.

### Plasmid constructs

Full-length coding sequences of Arabidopsis *AtDCP1*, *AtDCP2*, *AtXRN4* or *AtPARN* and *N*. *benthamiana NbDCP1*, *NbDCP2*, *NbXRN4* or *NbPARN* were retried from public domains and obtained by RT-PCR. The amplified fragments were subcloned into pDONR221 entry vector and confirmed by DNA sequencing, and then transferred into different gateway-compatible vectors, including pGADT7-DEST (AD), pGBKT7-DEST (BD) [51], pEarleyGate-101 (101, C-terminal YFP), pEarleyGate-102 (102, C-terminal CFP), pEarleyGate-104 (104, N-terminal YFP) [52], pBA-FLAG-4myc-DC (pBA, N-terminal FLAG-4×Myc) [53], pEarleygate201-YN [C-terminal YN (YN for the N-terminal half of YFP)] or pEarleygate202-YC [C-terminal YC (YC for the C-terminal half of YFP) [51], to generate corresponding expression vectors. Gateway-compatible vectors (AD/BD/101/102/104/pBA/YN/YC) containing TuMV P1/HC-Pro/P3 /P3N-PIPO/6K1/CI/6K2/VPg/Pro/NIb/CP were described previously [36,54,55]. An RNAi construct with an inverted repeat sequence of *NbDCP1* separated by an *Arabidopsis* intron was produced by overlapping PCR. A fragment of the *NbDCP1* sense sequence was amplified using primers A-NbDCP1-F and A-Intron+NbDCP1-R, and primers B-NbDCP1+Intron-F and B-Intron-BamHI-R. The overlapping products were cloned into pCHF3 between the *Sac*I and *Bam*HI sites to produce pCHF3-35S:NbDCP1-intron. The corresponding antisense NbDCP1 fragment was amplified using primers C-NbDCP1-F and C-NbDCP1-R and subsequently cloned into pCHF3-35S:NbDCP1-intron between the *Bam*HI and *Sal*I sites to produce the RNAi construct pCHF3-35S:NbDCP1. The similar strategy was used to generate the RNAi constructs of *GUS*, *NbDCP1*, *NbCP2*, *NbXRN4* and *NbPARN*. A partial fragment of *NbDCP1*, N*bDCP2*, *NbXRN4* or *NbPARN* was cloned into pTRV2 vector [56] to construct a TRV-based recombinant VIGS vector containing *NbDCP1*, N*bDCP2*, *NbXRN4* or *NbPARN*.

The coding sequence of full-length (1–711 bp), or N-terminal (1–340 bp) of mgfp5-ER was cloned into pCHF3 to generate pCHF3-35S-GFP or pCHF3-35S-GF. The antisense fragment of mgfp5-ER (N-terminal, 1–340 bp) was cloned into pCHF3-35S-GFP to generate pCHF3-35S-dsGF. The detailed primers and restriction endonuclease sites were given in **S1 Table**. pCHF3:P19 and PTGS suppression assays were described previously [35,41,57,58].

GenBank accession numbers for the genes analyzed in this study are as follows: NbDCP1 (KY402210), NbDCP2 (KY402211), NbXRN4 (KY402212), NbPARN (KY402213), AtDCP1 (NM_113710), AtDCP2 (NM_131203375), AtXRN4 (AF286718) and AtPARN (AB223028).

### Agroinfiltration and viral inoculation

*Agrobacterium*-mediated transient expression assays and viral infections in Wt *N*. *benthamiana* and H2B or dsRDR6 transgenic *N*. *benthamiana* plants were performed essentially as described [58,59]. The negative control plants were infiltrated with *Agrobacterium* cultures harboring an empty vector. The PTGS suppression assay and TuMV infection assay were repeated at least three times.

For the TRV-VIGS assay, *Agrobacterium* cultures harboring pTRV1 and one of the pTRV2 vectors including TRV2-GUS (control), TRV2-NbDCP1, TRV2-NbDCP2, TRV2-NbXRN4 and TRV2-NbPARN were mixed at a 1:1 ratio before infiltration.

### Y2H, BiFC, and subcellular localization

Y2H, BiFC and subcellular localization experiments were performed essentially as described [27,55,59].

### RNA analysis

For RNA blot analysis, total RNA was extracted from the infiltrated leaf patch, or virus-infected leaves with Trizol. High molecular weight (HMW) and low molecular weight (LMW) RNA analyses were conducted using 20 and 50 μg total RNA, respectively. HMW RNA was transferred to Hybond N^+^ nylon membranes (Amersham Pharmacia Biotech) by capillary transfer. Membranes were hybridized at 45°C to specific probes labeled with digoxigenin (Roche). For siRNA blotting, LMW RNAs were enriched from total RNA, transferred to a Hybond-N^+^ membrane and hybridized for detection of siRNAs as described previously [41,57,58]. Blotted membranes were visualized by chemiluminescence according to the manufacturer’s manual (ECL; GE Healthcare).

For qRT-PCR analysis, total RNA was isolated using the RNeasy Plant Mini Kit (Qiagen) and treated with DNase I (Thermo Fisher Scientific) following the manufacturer's instructions. cDNA synthesized from reverse transcription of RNA samples was used to determine the mRNA levels of target genes as well as for quantification of TuMV accumulation levels. The procedures of cDNA synthesis and qRT-PCR assays were described previously [35,55]. *NbActin* or *AtActinII* was used as an internal control for *N*. *benthamiana* and Arabidopsis, respectively. All primer information used in qRT-PCR was given in **S1 Table**.

### Protein analysis

Total protein was extracted from infiltrated leaf patches or TuMV-infiltrated or systemically infected *N*. *benthamiana* leaves as described previously [60]. Immunoblotting was performed with rabbit GFP polyclonal antibodies (ab6556, Abcam) or Myc polyclonal antibodies (ab9106, Abcam), followed by goat anti-rabbit (ab6721) secondary antibody conjugated to horseradish peroxidase (Abcam). Blotted membranes were washed thoroughly and visualized by chemiluminescence.

## Supporting information

S1 Table Primers used in this study (5'-3'). (DOCX)

S1 Fig The positive control of BiFC assays. P3N-PIPO and CI serve as a positive control for the protein-protein interaction assay in H2B transgenic *N*. *benthamiana* leaves at 32 hpi. The YFP halves (YN and YC) were fused with P3N-PIPO and CI. The interaction of P3N-PIPO and CI brought the split YFP halves in close proximity to restore yellow fluorescence (green). H2B-RFP is indicated by red. Bars = 25 μm.
(TIF)

S2 Fig Expression of dsGUS failed to suppress S-PTGS. (**A**) GFP fluorescence in *N*. *benthamiana*. Leaf patches were agroinfiltrated with three expression vectors including 35S-GFP, 35S-GF and one of the following vectors: an empty vector (Vec), dsGUS, dsNbXRN4, and P19. The representative picture was taken at 6 dpi under UV light. (**B**) Relative accumulation of *GFP* mRNAs analyzed by specific qRT-PCR in the infiltrated leaves shown in (A) at 6 dpi. *NbActin* serves as an internal standard. Values represent the mean ± SD (n = 3). Double asterisks indicate a highly significant difference compared to 35S-GFP+35S-GF+Vec (*P* < 0.01, Student’s *t* test).
(TIF)

S3 Fig Knock-down of *NbDCP1*, *NbDCP2*, *NbXRN4*, or *NbPARN* significantly enhances *GFP* expression in dsRDR6 transgenic *N*. *benthamiana* plants. (**A**) GFP fluorescence in RDR6-deficient *N*. *benthamiana*. Leaf patches were agroinfiltrated with three expression vectors including 35S-GFP, 35S-GF and one of the following vectors: an empty vector (Vec) as a control, dsNbDCP1, dsNbDCP2, dsNbXRN4, and dsNbPARN. The representative picture was taken at 7 dpi under UV light. (**B**) Relative accumulation of *GFP* mRNAs analyzed by specific qRT-PCR in the infiltrated leaves shown in (A) at 7 dpi. *NbActin* serves as an internal standard. Each mean value was based on three independent experiments (n = 3 samples). Values represent the mean ± SD. Double asterisks indicate a highly significant difference compared to 35S-GFP+35S-GF+Vec/dsRDR6 (*P* < 0.01, Student’s *t* test).
(TIF)

S4 Fig Overexpression of *NbDCP1*, *NbDCP2*, *NbXRN4*, or *NbPARN* reduces TuMV RNA accumulation. **(A)** qRT-PCR analyses of TuMV RNA levels. In this experiment, a very lower concentration of agrobacterium cultures (OD_600_ = 0.05) harboring TuMV or TuMV-ΔGDD was used. RNA was extracted from the infiltrated patches with TuMV-GFP or TuMV-ΔGDD and one of the following vectors: Vec, NbDCP1, NbDCP2, NbXRN4 or NbPARN at 3 dpi. Each value was normalized against *NbActin* transcripts in the same sample. Error bars represent SD (n = 3). Double asterisks indicate a highly significant difference compared to the treatment of Vec (*P* < 0.01, Student’s *t* test). **(B)** Accumulation of TuMV siRNAs in the infiltrated patches as described in (A) at 3 dpi. Northern blotting was performed using DIG-labeled DNA probes complementary to the TuMV genome. U6 serves as a loading control for siRNA blot, respectively.
(TIF)

S5 Fig Knock-down of *NbDCP1*, *NbDCP2*, *NbXRN4*, or *NbPARN* enhances TuMV infection. Accumulation of GFP protein and TuMV siRNAs in *N*. *benthamiana* leaves co-infiltrated with TuMV-GFP and one of the following vectors including an empty vector (Vec), NbDCP1, dsNbDCP1, dsNbDCP2, dsNbXRN4 and dsNbPARN at 4 dpi. Coomassie blue staining of the large subunit of Rubisco and U6 serve as a loading control for immunoblot, mRNA blot and siRNA blot, respectively. The values of GFP siRNAs/U6 were quantified by ImageJ software and then were normalized against the mean value corresponding to the Vec treatment, which was set to 1.00.
(TIF)

S6 Fig The phenotypes and RNA expression levels of *NbDCP1*, *NbDCP2*, *NbXRN4*, or *NbPARN*-deficient plants. **(A)** The phenotypes of TRV-GUS, TRV-NbDCP1, NbDCP2, NbXRN4, or TRV-NbPARN -treated plants at 14 dpi. A cDNA fragment of *NbDCP1*, *NbDCP2*, *NbXRN4*, or *NbPARN* was cloned into RNA2 of the TRV VIGS vector. *N*. *benthamiana* plants at the 4–5 leaf stage were infiltrated with *Agrobacterium* cultures carrying pTRV1 and pTRV2-GUS, or pTRV1 and TRV-NbDCP1, TRV-NbDCP2, TRV-NbXRN4, or TRV-NbPARN. **(B)** Silencing of target genes was confirmed in newly emerged leaves 14 dpi by qRT-PCR. **, *P* < 0.01, student’s *t* test.
(TIF)

S7 Fig Genotyping analysis of Arabidopsis *dcp1*, *dcp2*, *xrn4 and parn* T-DNA insertion lines. (**A, B, C, D**) Confirmation for homozygous *dcp1* (A), heterozygous *dcp2* (B), homozygous *xrn4* (C) and heterozygous *parn* (D) T-DNA insertion lines. PCR was conducted using genomic DNA from *dcp1* (SALK_014408C), *dcp2* (SALK_00519), *xrn4* (SALK_014209), *parn* (Salk_072627) mutant and wild type (Col-0) Arabidopsis plants. Gene-specific primers (LP+RP) were used to detect the wild type DNA from Col-0 plants. A T-DNA specific primer and a gene-specific primer (LBb1.3+RP) were used to amplify a single PCR fragment which represented the inserted DNA. LP, left genomic primer; RP, right genomic primer; LBb1.3, left border primer of the T-DNA insertion.
(TIF)

S8 Fig Deficiency of AtDCP1, AtDCP2, AtXRN4 or AtPARN facilitates TuMV infection. (**A**) Symptoms of TuMV-infected wild type (Col-0), *dcp1*, *dcp2*, *xrn4* and *parn* mutant Arabidopsis plants. Images were taken at 16 dpi. Mock means that plants were inoculated with buffer and TuMV means that plants were inoculated with TuMV infectious clones. (**B**) Quantification of TuMV RNA levels by qRT-PCR. RNA was extracted from TuMV systemically infected leaves at 16 dpi. The values are shown as means ± SD (n = 3) relative to Col-0 and *AtActinII* were used as the internal reference. Data were analyzed using Student’s t test (*, *P* < 0.05; **, *P* < 0.01).
(TIF)

S9 Fig The interaction analysis between NbDCP2 and VPg. The YFP halves (YN and YC) were fused with NbDCP2 and VPg. The interaction of NbDCP2 and VPg was present in the nucleus (green) in H2B transgenic *N*. *benthamiana* leaves at 32 hpi. H2B-RFP is indicated in red. Bars = 10 μm.
(TIF)

S10 Fig Expression analysis of AtXRN4, HC-Pro and HC-Pro/XRN4 transgenic Arabidopsis plants. (**A**) qRT-PCR analysis of *AtXRN4* expression in Col-0 and transgenic plants carrying 35S:Myc-AtXRN4-1, 35S:Myc-AtXRN4-2, 35S:HC-Pro-CFP-1/35S:Myc-AtXRN4-1 or 35S:HC-Pro-CFP-1/35S:Myc-AtXRN4-2. (**B**) Detection of Myc-AtXRN4 in 35S:Myc-AtXRN4, and 35S:HC-Pro-CFP/35S:Myc-AtXRN4 transgenic plants by immunoblotting. (**C**) qRT-PCR analysis of *HC-Pro* expression in Col-0 and transgenic plants carrying 35S:Myc-AtXRN4-1, 35S:Myc-AtXRN4-2, 35S:HC-Pro-CFP-1/35S:Myc-AtXRN4-1 or 35S:HC-Pro-CFP-1/35S:Myc-AtXRN4-2. (**D**) Confirmation of the protein expression from 35S:HC-Pro-CFP and 35S:HC-Pro-CFP/35S:Myc-AtXRN4 transgenic Arabidopsis plants by Western blot. Anti-Myc (B) or anti-GFP (D) polyclonal antibodies were used, respectively. Coomassie brilliant blue (CBB)-stained Rubisco large subunit was used as a loading control. 35S:HC-Pro-CFP/35S:Myc-AtXRN4 plants were obtained by genetic crosses between 35S:HC-Pro-CFP-1 and 35S:Myc-AtXRN4-1 *Arabidopsis* plants. T2 generation plants were used in the above experiments.
(TIF)
