# Supplementary material for: RNA decay is an antiviral defense in plants that is counteracted by viral RNA silencing suppressors
Source: PLoS Pathog. 2018 Aug 3;14(8):e1007228. doi: 10.1371/journal.ppat.1007228 (PMC6101400; doi:10.1371/journal.ppat.1007228)
Supplement: S1 Table — (DOCX) [file ppat.1007228.s001.docx]

**Supplementary Table S1: Primers used in this study (5'-3')**

| qPCR-TuMV-CP-F | TGGCTGATTACGAACTGACG |
| --- | --- |
| qPCR-TuMV-CP-R | CTGCCTAAATGTGGGTTTGG |
| qPCR-TuMV-HC-Pro-F | GGAGCCAACTTCTGGAAAG |
| qPCR-TuMV-HC-Pro-R | GGTTATCTTTCCGCATGGGAACA |
| qPCR-AtActinII-F | CACCACAACAGCAGAGCGGGA |
| qPCR-AtActinII-R | TCCCACAAACGAGGGCTGGA |
| qPCR-NbActin-F | AAAGACCAGCTCATCCGTGGAGAA |
| qPCR-NbActin-R | TGTGGTTTCATGAATGCCAGCAGC |
| NbDCP1-gn-F | GGGGACAAGTTTGTACAAAAAAGCAGGCTTCATGTCACAGAACGGAAAATTAATGC |
| NbDCP1-gn-R | GGGGACCACTTTGTACAAGAAAGCTGGGTCTGAATGATGAGCATTTAGCACTG |
| TRV-NbDCP1-F | CGGGATCCTGGACCAGAACAGCACCAAGC |
| TRV-NbDCP1-R | CCGCTCGAGCTTCTTGGGAAGCATTTCGATAC |
| qPCR-NbDCP1-F | CAGTTTATTGTTATGAACCGAAGAAATACAG |
| qPCR-NbDCP1-R | ACTTTTGACTTGGTAGGCACCTTGG |
| A-NbDCP1-F | GGGGTACCTGGACCAGAACAGCACCAAGC |
| A-Intron+NbDCP1-R | AAATCAGACTTACAACGTACCTTGGAATATGCACCG |
| B-NbDCP1+Intron-F | CGGTGCATATTCCAAGGTACGTTGTAAGTCTGATTT |
| B-Intron-BamH-R | CGGGATCCGCTCTATCTGCTGGGTCCAAATC |
| C-NbDCP1-F | CGGGATCCACCTTGGAATATGCACCG |
| C-NbDCP1-R | GCGTCGACGTCACAGAACGGAAAATTAATGCCG |
| NbDCP2-gn-F | GGGGACAAGTTTGTACAAAAAAGCAGGCTTCATGTCAGGGCTTCATCGATCTTC |
| NbDCP2-gn-R | GGGGACCACTTTGTACAAGAAAGCTGGGTCAGAAGAGAAGCCAGCTTCCATTG |
| TRV-NbDCP2-F | CGGGATCCGAACTCCTTGACGATCTTTGC |
| TRV-NbDCP2-R | CCGCTCGAGCCTTTACTAGCAAGCACCTTTC |
| qPCR-NbDCP2-F | ACATATTCAAGGACTTTACCTCTTACAAGG |
| qPCR-NbDCP2-R | GATACATCAAATCCTGTTTCTTCCAAGAC |
| A-NbDCP2-F | GGGGTACC GAACTCCTTGACGATCTTTGC |
| A-Intron+NbDCP2-R | AAATCAGACTTACAACGTCTTCTCTAACTGCACA |
| B-NbDCP2+Intron-F | TGTGCAGTTAGAGAAGACGTTGTAAGTCTGATTT |
| C-NbDCP2-F | CGGGATCCCTTCTCTAACTGCACA |
| C-NbDCP2-R | GCGTCGACGAACTCCTTGACGATCTTTGC |
| NbXRN4-gn-F | GGGGACAAGTTTGTACAAAAAAGCAGGCTTCATGGGAGTACCAGCATTTTATAGG |
| NbXRN4-gn-R | GGGGACCACTTTGTACAAGAAAGCTGGGTCTTGATGTGTTCCTGTTTCTTCGG |
| TRV-NbXRN4-F | CGGGATCCAACCCTAATGGCATGGAATTTGATAAC |
| TRV-NbXRN4-R | CCGCTCGAGGCAATAAACTAGCAGCCTCCATC |
| qPCR-NbXRN4-F | GGAGGCTGCTAGTTTATTGCC |
| qPCR-NbXRN4-R | GTAGGACATAATTTTGTGTTCACCC |
| A-NbXRN4-F | GGGGTACCAACCCTAATGGCATGGAATTTGATAAC |
| A-Intron+NbXRN4-R | AAATCAGACTTACAACGTCTGAAGAGCTACTGC |
| B-NbXRN4+Intron-F | GCAGTAGCTCTTCAGACGTTGTAAGTCTGATTT |
| C-NbXRN4-F | CGGGATCCCTGAAGAGCTACTGC |
| C-NbXRN4-R | GCGTCGACAACCCTAATGGCATGGAATTTGATAAC |
| AtXRN4-gn-F | GGGGACAAGTTTGTACAAAAAAGCAGGCTTCATGGGAGTACCGGCGTTCTAC |
| AtXRN4-gn-R | GGGGACCACTTTGTACAAGAAAGCTGGGTCCAAGTTTGCACCTCGATGACTTG |
| qPCR-AtXRN4-F | AGAGCTGCAAAGGACGCAGCAG |
| qPCR-AtXRN4-R | AACATATCTCCAGCCAGGGTTATGG |
| NbPARN-gn-F | GGGGACAAGTTTGTACAAAAAAGCAGGCTTCATGAAGAAACAGTGTATGTTG |
| NbPARN-gn-R | GGGGACCACTTTGTACAAGAAAGCTGGGTCATTGCTTGCCAGAGCTTCATTG |
| TRV-NbAPRN-F | CGGGATCCACTGCTGAGGACTATGTGTCTTC |
| TRV-NbAPRN-R | CCGCTCGAGCTAGATGATTGCACGCCTGTGC |
| qPCR-NbPARN-F | ATCCTTGAACTGGAACTCAGGGTCC |
| qPCR-NbPARN-R | CCCAGTACTTAGATCAATGATGTCTCC |
| A-NbPARN-F | GGGGTACCACTGCTGAGGACTATGTGTCTTC |
| A-NbPARN-intr-R | AAATCAGACTTACAACGTCTAGATGATTGCACGCCTGTGC |
| B-NbPARN-intr-F | GCACAGGCGTGCAATCATCTAGACGTTGTAAGTCTGATTT |
| C-NbPARN-BamHI-F | CGGGATCCCTAGATGATTGCACGCCTGTGC |
| C-NbPARN-salI-R | GCGTCGACACTGCTGAGGACTATGTGTCTTC |
| 35S-F | CGCAAGACCCTTCCTCTATATAAGGAA |
| LBb1.3-F | [ATTTTGCCGATTTCGGAAC](http://signal.salk.edu/pBIN-pROK2.txt-new) |
| LP-xrn4-209-F | AGGTGTATGCTCTTGGCAATG |
| RP-xrn4-209-R | AACTGCCATGAAAACTGATGG |
| LP-dcp1-40820-F | CCTTGGAGTCAAAGGTGAATG |
| RP-dcp1-40820-R | TTGAGACTAACCCATTGGCTG |
| LP-dcp2-519-F | TTTGCTATTCTTTGACCTCCG |
| RP-dcp2-519-R | CTCCTTGATGATCTTTGCAGG |
| LP-parn-627-F | AGCTGTTGAAGGAAGAGGACC  mAtPARN-R ACTCACATTTGATCCACCAGC |
| RP-parn-627-R | ACTCACATTTGATCCACCAGC |
| GFP-BamHF | CGGGATCCATGAGTAAAGGAGAAGAACTTTTC |
| GFP-SalR | ACGCGTCGACTTTTTGTATAGTTCATCCATG |
| GFPN-SalR | ACGCGTCGACCAAACTTGACTTCAGCAC |
| FG-A-KpnI-F | GGGGTACCCAAACTTGACTTCAGCAC |
| A-inr-FG-R | AAATCAGACTTACAACGTATGAGTAAAGGAGAAGAACTTTTC |
| B-FG-intr-F | GAAAAGTTCTTCTCCTTTACTCATTACGTTGTAAGTCTGATTT |
| qPCR-AtEBF2-F | GTTTGTCTGAGATCGCACGGTCATG |
| qPCR-AtEBF2-R | ACGTGGGCAGCTCCTGATAGAGATAG |
| qPCR-AtRAP2.4-F | ATGGCAGCTGCTATGAATTTGTACAC |
| qPCR-AtRAP2.4-R | CGTATGAAAACGGTTGGGTCAAGAACG |
| qPCR-AtAtNMT-F | ATGGCTCACAGTCACACTAATGGCGC |
| qPCR-AtAtNMT-R | CTCTAGCACTGTTGTCCCTTCAATAGG |
| qPCR-GFP-F | AGCGGCACGACTTCTTCAAGAG |
| qPCR-GFP-R | GTTGTGGGAGTTGTAGTTGTATTCC |
| 5'Digoxigenin probed-TuMV oligo-1 | AGCATTCAAGCAATCAAAGATTCTCAAATCTTTCAT |
| 5'Digoxigenin probed-TuMV oligo-2 | ATGCCATTGCGATCCACCACCGTCACCACAGT |
| 5'Digoxigenin probed-TuMV oligo-3 | GCTTTCTCGCATACCGTAGTGACAATCGCGAG |
| 5'Digoxigenin probed-TuMV oligo-4 | AGCGAACAGTCACTATCATAGATACACTAATCGCTG |
| 5'Digoxigenin probed-TuMV oligo-5 | AAGGGTAGGACACGTGGTATCGGACACAAAAACA |
| 5'Digoxigenin probed-TuMV oligo-6 | AAACCTCGATCATCTAATCCTATACACGCCGGAGC |
| 5'Digoxigenin probed-U6 | TTGCGTGTCATCCTTGCGCAGG |
| 5'Digoxigenin probed-G-1 | ATGAGTAAAGGAGAAGAACT |
| 5'Digoxigenin probed-G-2 | CCAATTCTTGTTGAATTAGATGGTG |
| 5'Digoxigenin probed-G-3 | TCTGTCAGTGGAGAGGGTGAAGGTGATGCAA |
| 5'Digoxigenin probed-F-1 | CTACCTGTTCCGTGGCCAACACTTG |
| 5'Digoxigenin probed-F-2 | CTACCTGTTCCATGGCCAACACT |
| 5'Digoxigenin probed-F-3 | TGTTCAATGCTTTTCAAGATACCC |
| 5'Digoxigenin probed-P-1 | AAGCAAAAGAACGGCATCAA |
| 5'Digoxigenin probed-P-2 | CATTATCAACAAAATACTCCAATTGGCGATG |
| 5'Digoxigenin probed-P-3 | CAATTGGCGATGGCCCTGTCC |

Gateway-compatible adaptor sequences and restriction endonuclease sites are underlined
