# Supplementary figures and images for: RNA decay is an antiviral defense in plants that is counteracted by viral RNA silencing suppressors
Source: PLoS Pathog. 2018 Aug 3;14(8):e1007228. doi: 10.1371/journal.ppat.1007228 (PMC6101400; doi:10.1371/journal.ppat.1007228)

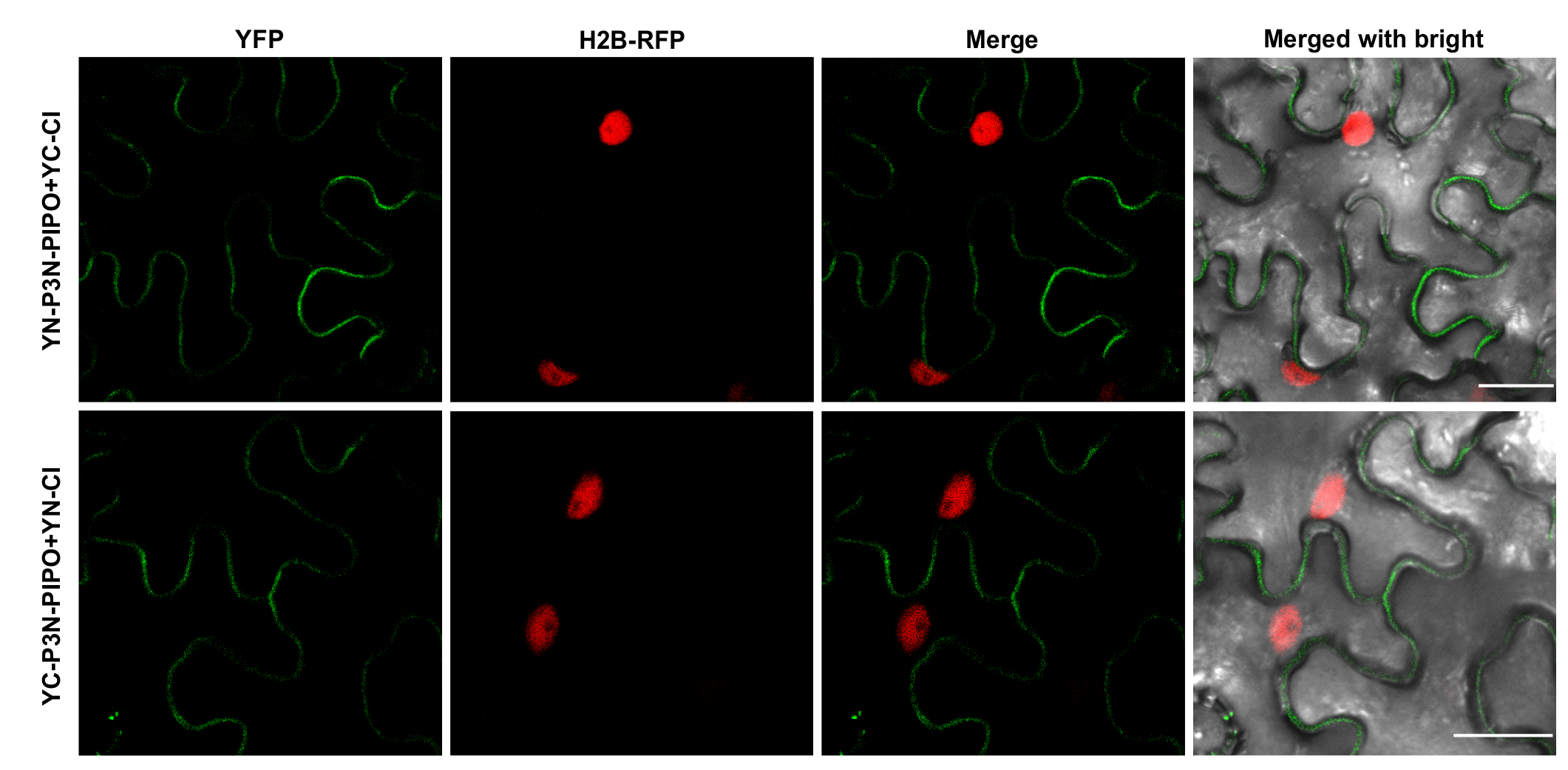

Supplement: S1 Fig — P3N-PIPO and CI serve as a positive control for the protein-protein interaction assay in H2B transgenic N. benthamiana leaves at 32 hpi. The YFP halves (YN and YC) were fused with P3N-PIPO and CI. The interaction of P3N-PIPO and CI brought the split YFP halves in close proximity to restore yellow fluorescence (green). H2B-RFP is indicated by red. Bars = 25 μm. (TIF) [file ppat.1007228.s002.tif]

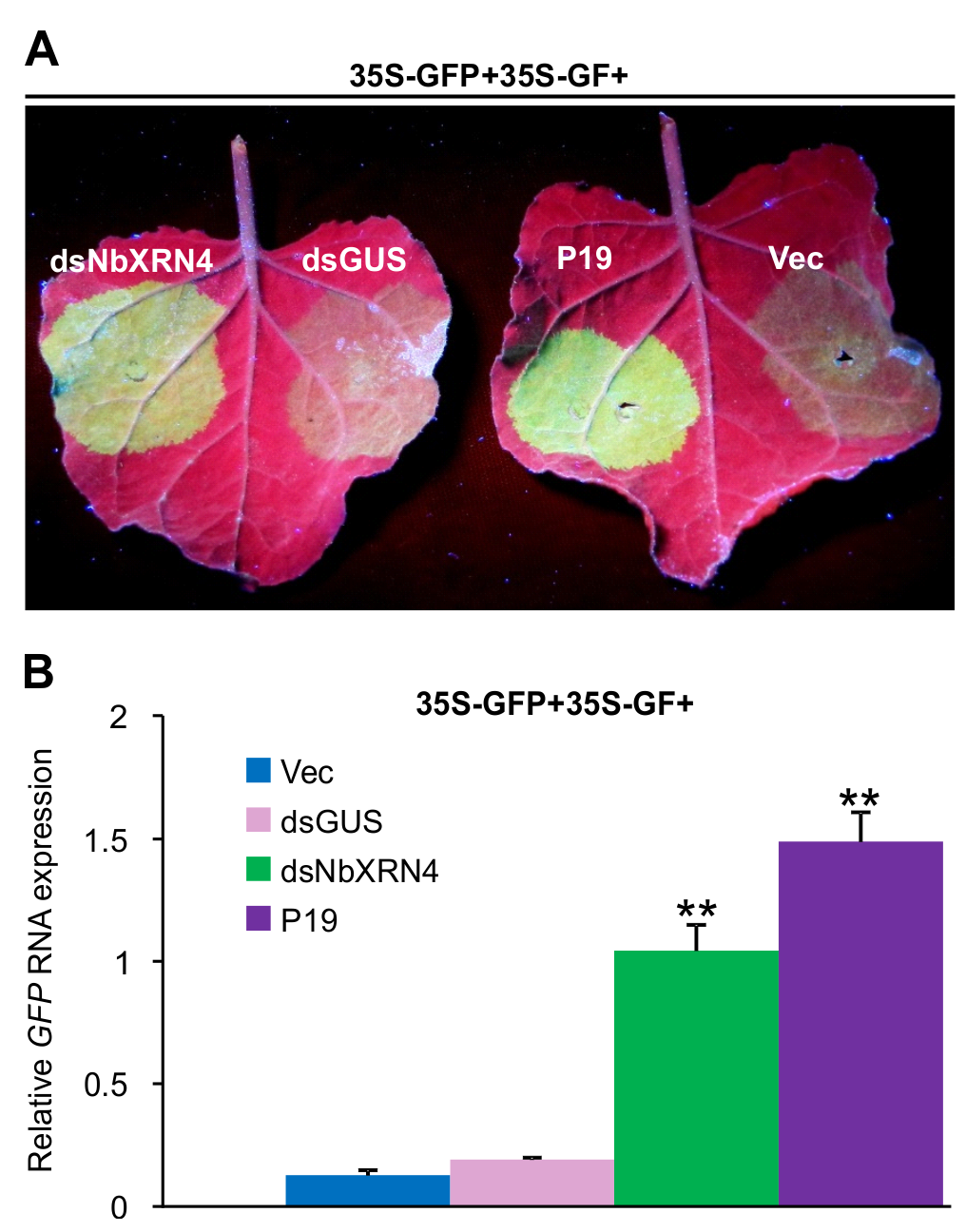

Supplement: S2 Fig — (A) GFP fluorescence in N. benthamiana. Leaf patches were agroinfiltrated with three expression vectors including 35S-GFP, 35S-GF and one of the following vectors: an empty vector (Vec), dsGUS, dsNbXRN4, and P19. The representative picture was taken at 6 dpi under UV light. (B) Relative accumulation of GFP mRNAs analyzed by specific qRT-PCR in the infiltrated leaves shown in (A) at 6 dpi. NbActin serves as an internal standard. Values represent the mean ± SD (n = 3). Double asterisks indicate a highly significant difference compared to 35S-GFP+35S-GF+Vec (P < 0.01, Student’s t test). (TIF) [file ppat.1007228.s003.tif]

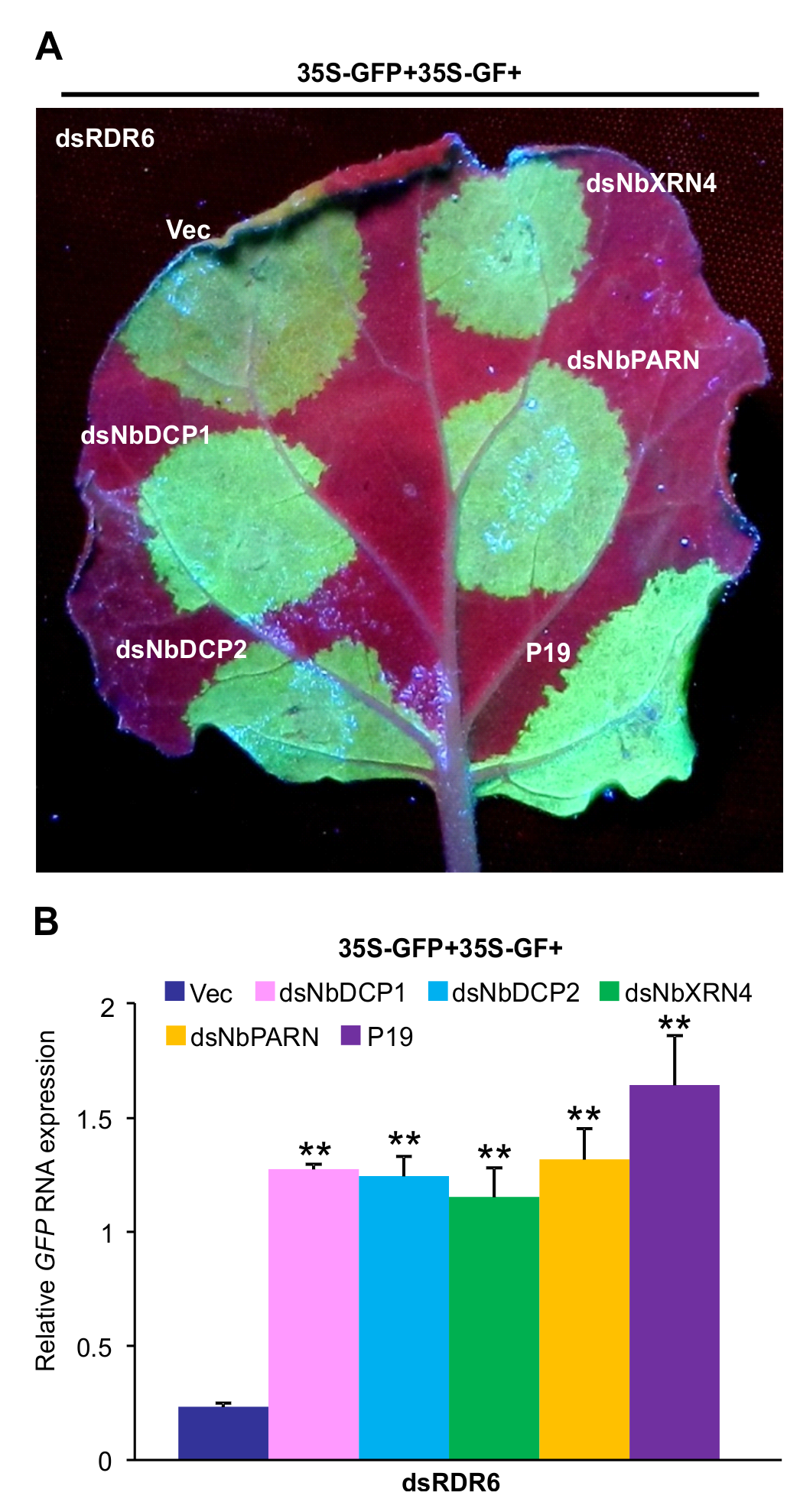

Supplement: S3 Fig — (A) GFP fluorescence in RDR6-deficient N. benthamiana. Leaf patches were agroinfiltrated with three expression vectors including 35S-GFP, 35S-GF and one of the following vectors: an empty vector (Vec) as a control, dsNbDCP1, dsNbDCP2, dsNbXRN4, and dsNbPARN. The representative picture was taken at 7 dpi under UV light. (B) Relative accumulation of GFP mRNAs analyzed by specific qRT-PCR in the infiltrated leaves shown in (A) at 7 dpi. NbActin serves as an internal standard. Each mean value was based on three independent experiments (n = 3 samples). Values represent the mean ± SD. Double asterisks indicate a highly significant difference compared to 35S-GFP+35S-GF+Vec/dsRDR6 (P < 0.01, Student’s t test). (TIF) [file ppat.1007228.s004.tif]

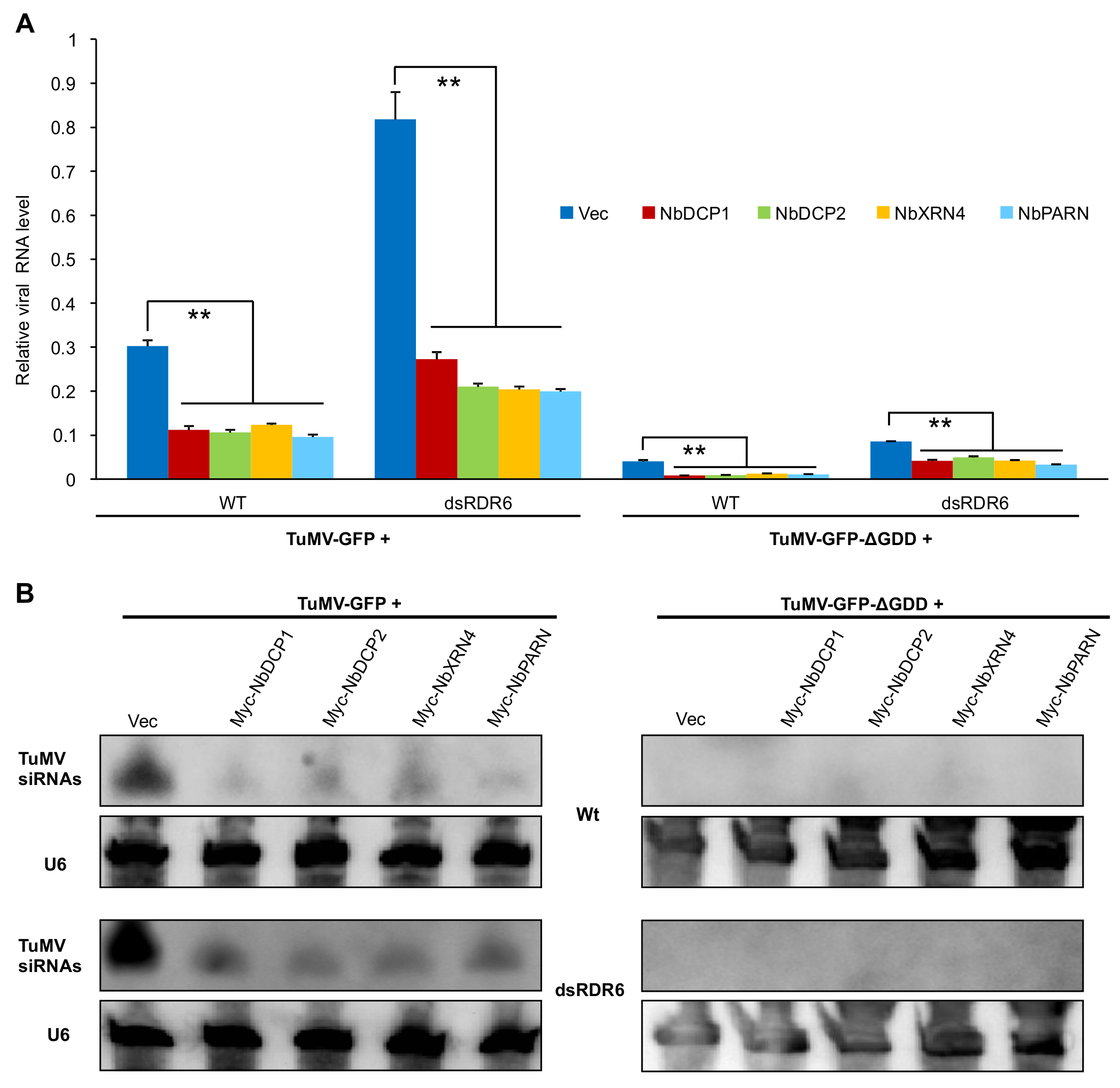

Supplement: S4 Fig — (A) qRT-PCR analyses of TuMV RNA levels. In this experiment, a very lower concentration of agrobacterium cultures (OD600 = 0.05) harboring TuMV or TuMV-ΔGDD was used. RNA was extracted from the infiltrated patches with TuMV-GFP or TuMV-ΔGDD and one of the following vectors: Vec, NbDCP1, NbDCP2, NbXRN4 or NbPARN at 3 dpi. Each value was normalized against NbActin transcripts in the same sample. Error bars represent SD (n = 3). Double asterisks indicate a highly significant difference compared to the treatment of Vec (P < 0.01, Student’s t test). (B) Accumulation of TuMV siRNAs in the infiltrated patches as described in (A) at 3 dpi. Northern blotting was performed using DIG-labeled DNA probes complementary to the TuMV genome. U6 serves as a loading control for siRNA blot, respectively. (TIF) [file ppat.1007228.s005.tif]

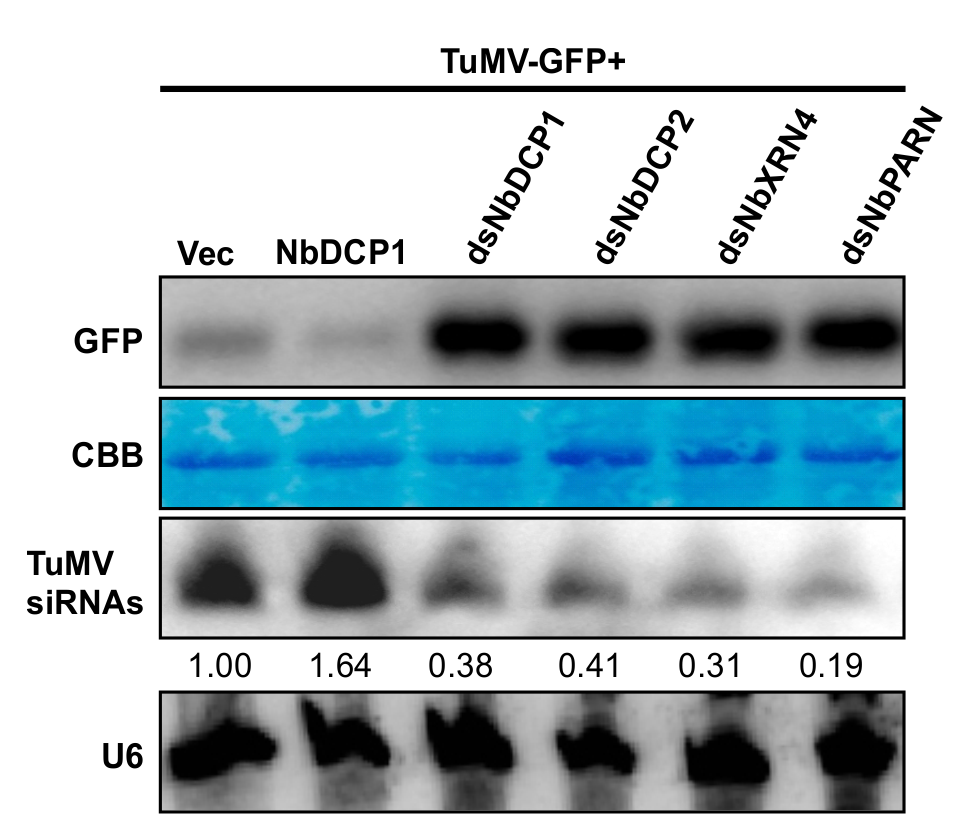

Supplement: S5 Fig — Accumulation of GFP protein and TuMV siRNAs in N. benthamiana leaves co-infiltrated with TuMV-GFP and one of the following vectors including an empty vector (Vec), NbDCP1, dsNbDCP1, dsNbDCP2, dsNbXRN4 and dsNbPARN at 4 dpi. Coomassie blue staining of the large subunit of Rubisco and U6 serve as a loading control for immunoblot, mRNA blot and siRNA blot, respectively. The values of GFP siRNAs/U6 were quantified by ImageJ software and then were normalized against the mean value corresponding to the Vec treatment, which was set to 1.00. (TIF) [file ppat.1007228.s006.tif]

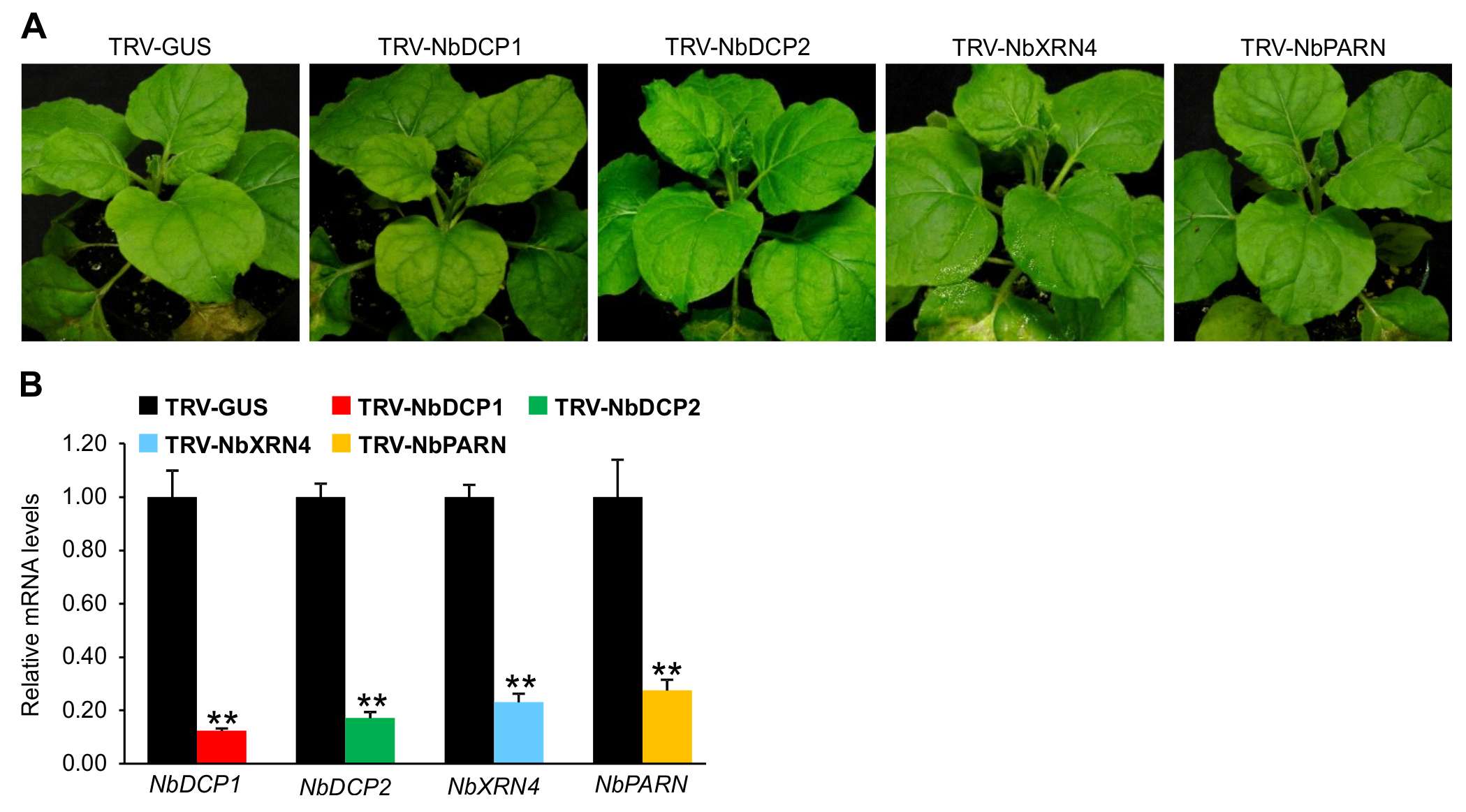

Supplement: S6 Fig — (A) The phenotypes of TRV-GUS, TRV-NbDCP1, NbDCP2, NbXRN4, or TRV-NbPARN -treated plants at 14 dpi. A cDNA fragment of NbDCP1, NbDCP2, NbXRN4, or NbPARN was cloned into RNA2 of the TRV VIGS vector. N. benthamiana plants at the 4–5 leaf stage were infiltrated with Agrobacterium cultures carrying pTRV1 and pTRV2-GUS, or pTRV1 and TRV-NbDCP1, TRV-NbDCP2, TRV-NbXRN4, or TRV-NbPARN. (B) Silencing of target genes was confirmed in newly emerged leaves 14 dpi by qRT-PCR. **, P < 0.01, student’s t test. (TIF) [file ppat.1007228.s007.tif]

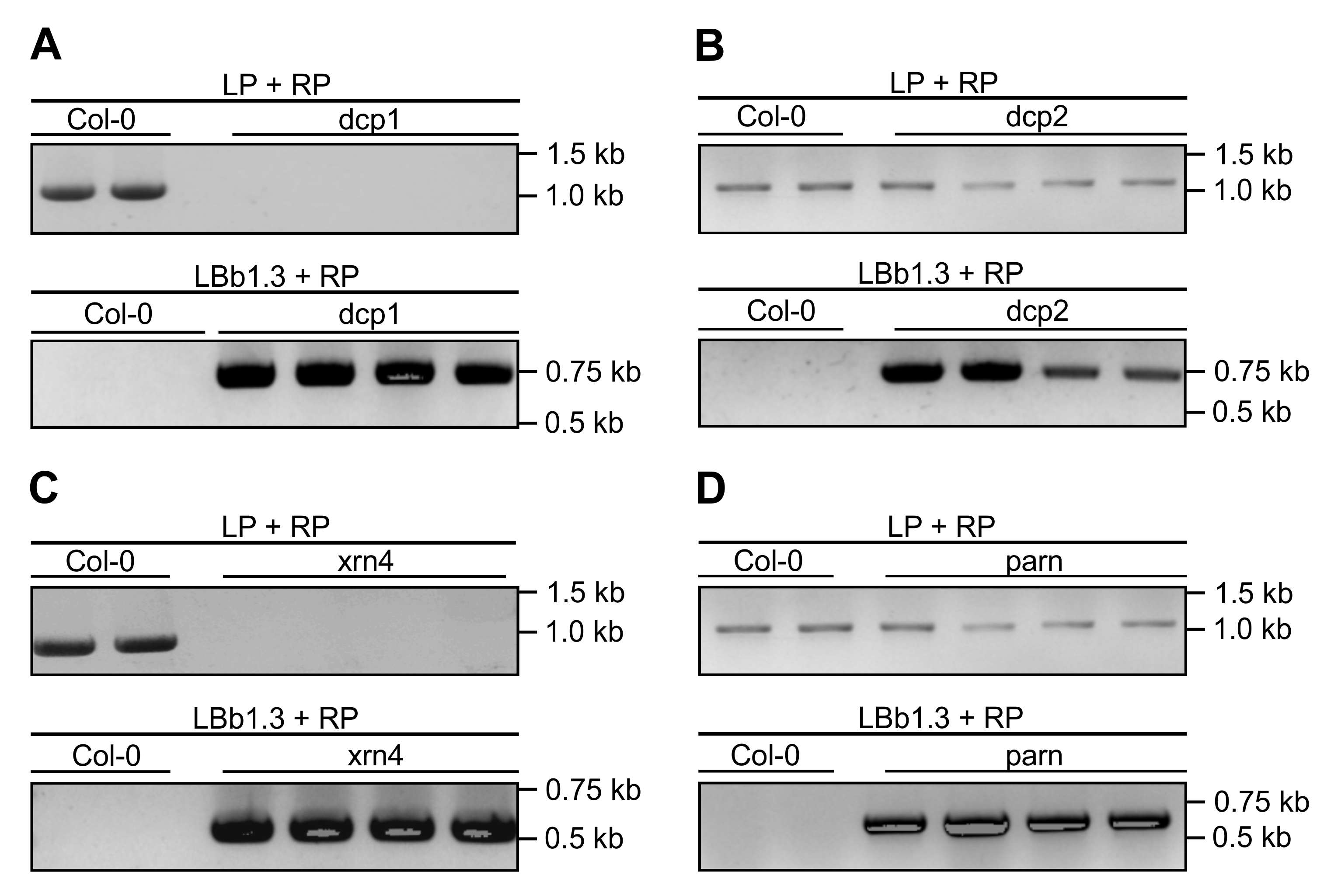

Supplement: S7 Fig — (A, B, C, D) Confirmation for homozygous dcp1 (A), heterozygous dcp2 (B), homozygous xrn4 (C) and heterozygous parn (D) T-DNA insertion lines. PCR was conducted using genomic DNA from dcp1 (SALK_014408C), dcp2 (SALK_00519), xrn4 (SALK_014209), parn (Salk_072627) mutant and wild type (Col-0) Arabidopsis plants. Gene-specific primers (LP+RP) were used to detect the wild type DNA from Col-0 plants. A T-DNA specific primer and a gene-specific primer (LBb1.3+RP) were used to amplify a single PCR fragment which represented the inserted DNA. LP, left genomic primer; RP, right genomic primer; LBb1.3, left border primer of the T-DNA insertion. (TIF) [file ppat.1007228.s008.tif]

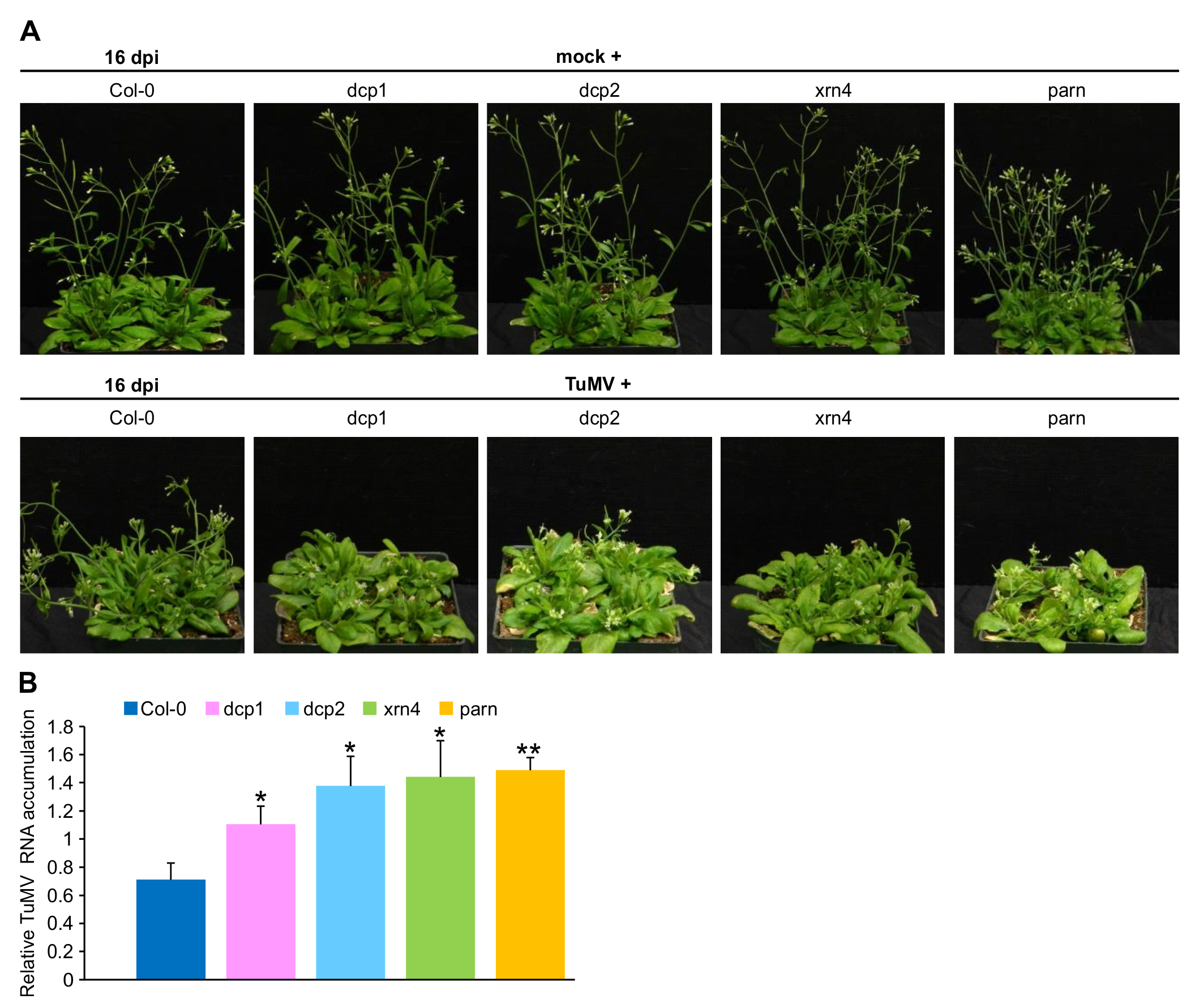

Supplement: S8 Fig — (A) Symptoms of TuMV-infected wild type (Col-0), dcp1, dcp2, xrn4 and parn mutant Arabidopsis plants. Images were taken at 16 dpi. Mock means that plants were inoculated with buffer and TuMV means that plants were inoculated with TuMV infectious clones. (B) Quantification of TuMV RNA levels by qRT-PCR. RNA was extracted from TuMV systemically infected leaves at 16 dpi. The values are shown as means ± SD (n = 3) relative to Col-0 and AtActinII were used as the internal reference. Data were analyzed using Student’s t test (*, P < 0.05; **, P < 0.01). (TIF) [file ppat.1007228.s009.tif]

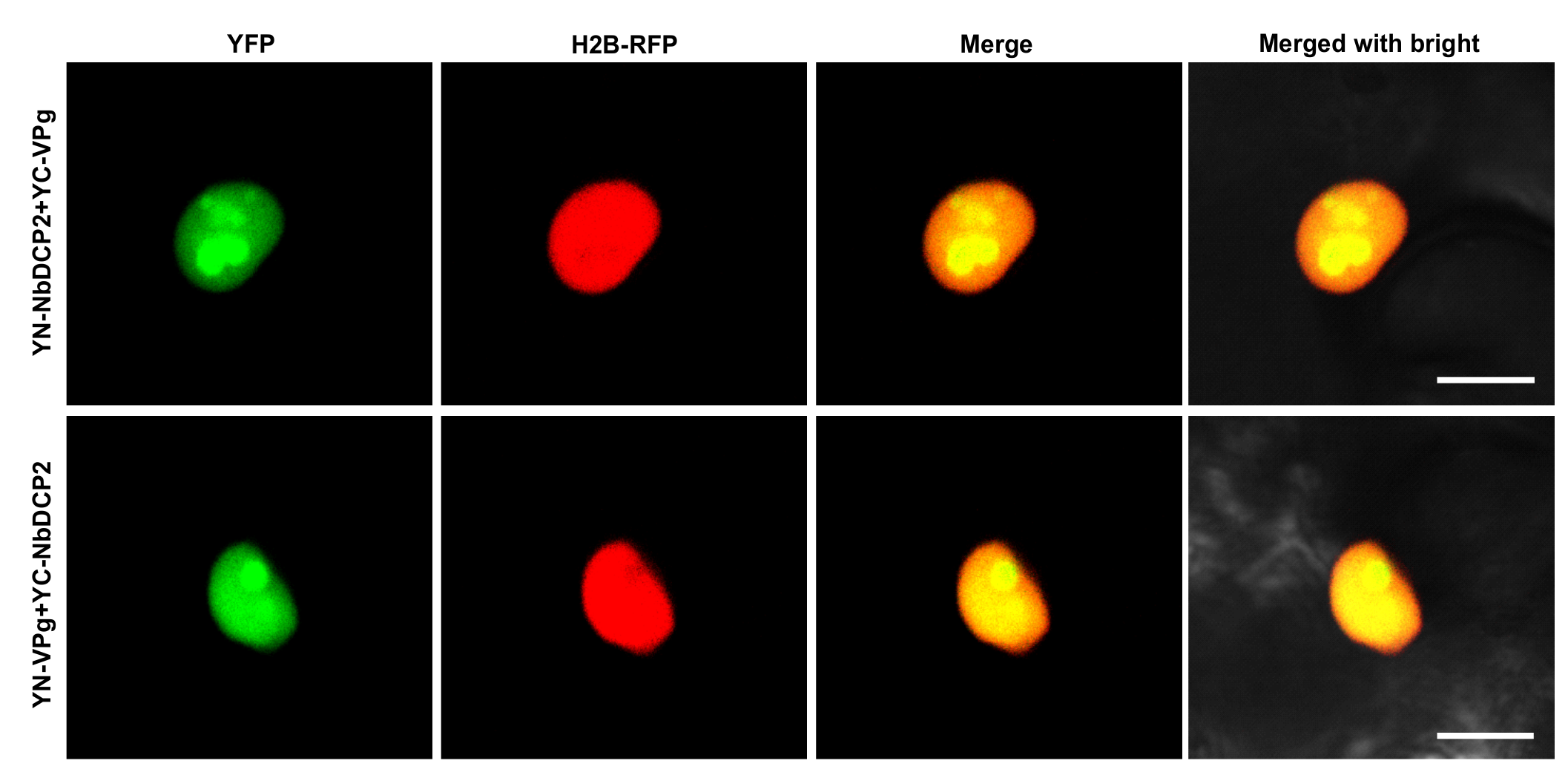

Supplement: S9 Fig — The YFP halves (YN and YC) were fused with NbDCP2 and VPg. The interaction of NbDCP2 and VPg was present in the nucleus (green) in H2B transgenic N. benthamiana leaves at 32 hpi. H2B-RFP is indicated in red. Bars = 10 μm. (TIF) [file ppat.1007228.s010.tif]

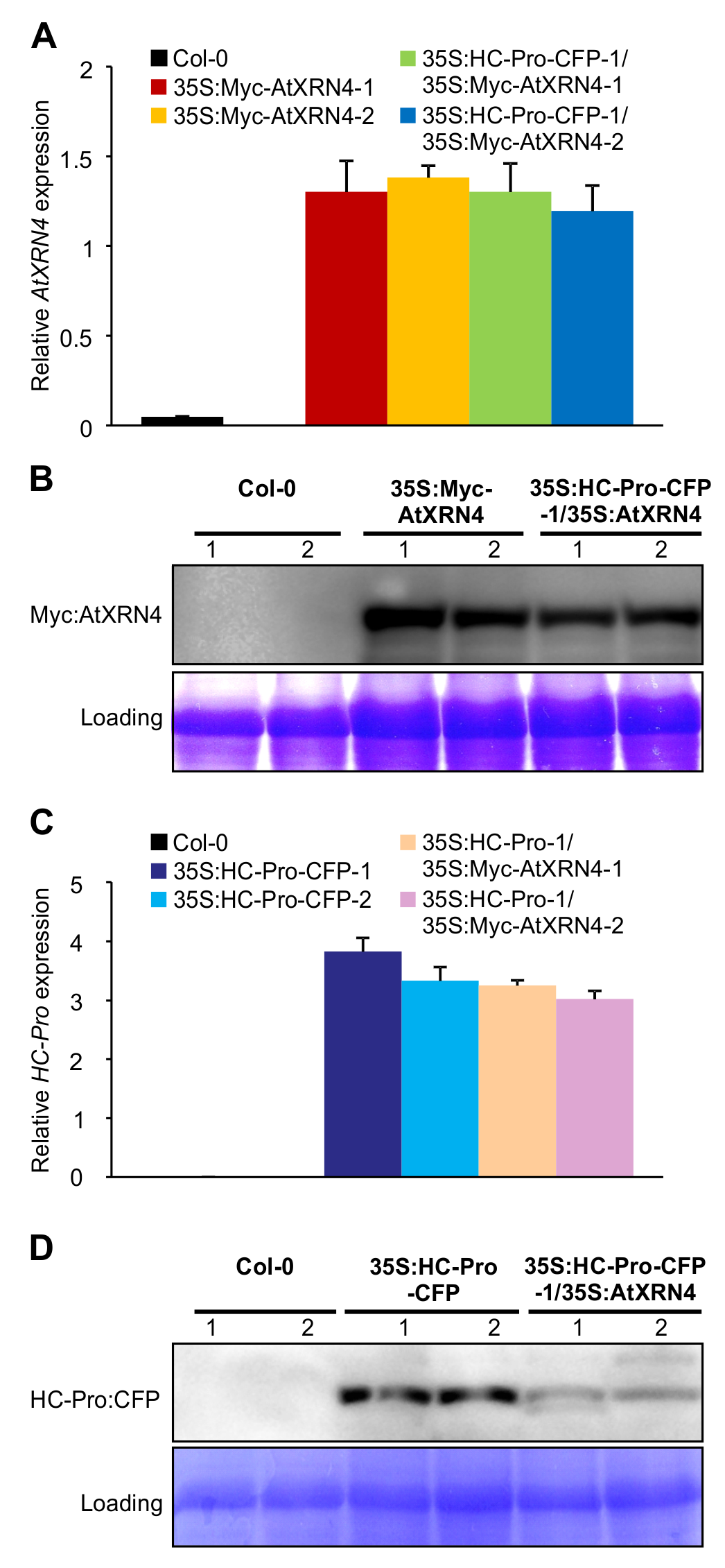

Supplement: S10 Fig — (A) qRT-PCR analysis of AtXRN4 expression in Col-0 and transgenic plants carrying 35S:Myc-AtXRN4-1, 35S:Myc-AtXRN4-2, 35S:HC-Pro-CFP-1/35S:Myc-AtXRN4-1 or 35S:HC-Pro-CFP-1/35S:Myc-AtXRN4-2. (B) Detection of Myc-AtXRN4 in 35S:Myc-AtXRN4, and 35S:HC-Pro-CFP/35S:Myc-AtXRN4 transgenic plants by immunoblotting. (C) qRT-PCR analysis of HC-Pro expression in Col-0 and transgenic plants carrying 35S:Myc-AtXRN4-1, 35S:Myc-AtXRN4-2, 35S:HC-Pro-CFP-1/35S:Myc-AtXRN4-1 or 35S:HC-Pro-CFP-1/35S:Myc-AtXRN4-2. (D) Confirmation of the protein expression from 35S:HC-Pro-CFP and 35S:HC-Pro-CFP/35S:Myc-AtXRN4 transgenic Arabidopsis plants by Western blot. Anti-Myc (B) or anti-GFP (D) polyclonal antibodies were used, respectively. Coomassie brilliant blue (CBB)-stained Rubisco large subunit was used as a loading control. 35S:HC-Pro-CFP/35S:Myc-AtXRN4 plants were obtained by genetic crosses between 35S:HC-Pro-CFP-1 and 35S:Myc-AtXRN4-1 Arabidopsis plants. T2 generation plants were used in the above experiments. (TIF) [file ppat.1007228.s011.tif]
